# Immunoinformatics-Aided Design of a Peptide Based Multiepitope Vaccine Targeting Glycoproteins and Membrane Proteins against Monkeypox Virus

**DOI:** 10.3390/v14112374

**Published:** 2022-10-27

**Authors:** Nahid Akhtar, Vikas Kaushik, Ravneet Kaur Grewal, Atif Khurshid Wani, Chonticha Suwattanasophon, Kiattawee Choowongkomon, Romina Oliva, Abdul Rajjak Shaikh, Luigi Cavallo, Mohit Chawla

**Affiliations:** 1 School of Bio-Engineering and Bio-Sciences, Lovely Professional University, Phagwara 144411, India; 2 Department of Research and Innovation, STEMskills Research and Education Lab Private Limited, Faridabad 121002, India; 3 Department of Biochemistry, Faculty of Science, Kasetsart University, 50 Ngam Wong Wan Rd, Chatuchak, Bangkok 10900, Thailand; 4 Department of Sciences and Technologies, University Parthenope of Naples, Centro Direzionale Isola C4, I-80143 Naples, Italy; 5 Physical Sciences and Engineering Division, Kaust Catalysis Center, King Abdullah University of Science and Technology (KAUST), Thuwal 23955-6900, Saudi Arabia

**Keywords:** monkeypox, monkeypox virus, immunoinformatics, epitope-based vaccine, orthopoxvirus, reverse vaccinology

## Abstract

Monkeypox is a self-limiting zoonotic viral disease and causes smallpox-like symptoms. The disease has a case fatality ratio of 3–6% and, recently, a multi-country outbreak of the disease has occurred. The currently available vaccines that have provided immunization against monkeypox are classified as live attenuated vaccinia virus-based vaccines, which pose challenges of safety and efficacy in chronic infections. In this study, we have used an immunoinformatics-aided design of a multi-epitope vaccine (MEV) candidate by targeting monkeypox virus (MPXV) glycoproteins and membrane proteins. From these proteins, seven epitopes (two T-helper cell epitopes, four T-cytotoxic cell epitopes and one linear B cell epitopes) were finally selected and predicted as antigenic, non-allergic, interferon-γ activating and non-toxic. These epitopes were linked to adjuvants to design a non-allergic and antigenic candidate MPXV-MEV. Further, molecular docking and molecular dynamics simulations predicted stable interactions between predicted MEV and human receptor TLR5. Finally, the immune-simulation analysis showed that the candidate MPXV-MEV could elicit a human immune response. The results obtained from these in silico experiments are promising but require further validation through additional in vivo experiments.

## 1. Introduction

Monkeypox is a zoonotic viral infection with a 3–6% fatality rate and is caused by the monkeypox virus (MPXV) [1,2]. Two subfamilies of the MPXV, namely West African and Central African, are known but the mortality rate is observed to be higher with the Central African subfamily of the virus [2]. Humans can be exposed to the MPXV through respiratory droplets, body fluids, lesions of the infected person, interactions with infected animals and contact with virus-contaminated fomites [3,4]. MPXV was isolated for the first time in Denmark from the lesions of infected cynomolgus macaques, monkeys imported from Singapore in 1958 [5]. Human infection with MPXV was first observed in an infant with smallpox-like symptoms [5]. Since then, human cases of monkeypox have been recorded in 11 Western and Central African countries such as Nigeria, Sierra Leone, and the Central African Republic [5,6,7]. The human transmission of MPXV could also be attributed to the end of the smallpox vaccination program, which provided cross-immunity against MPXV [8]. Recently, there has been a multi-country outbreak of human monkeypox cases [9].

Although monkeypox is self-limiting, people with comorbidities and compromised immunity, pregnant women, and the pediatric population are at higher risk. The protection from MPXV in humans could be provided by the smallpox vaccine [5]. However, the smallpox vaccination program has been stopped after the eradication of smallpox in 1980 [10]. ACAM2000, a live vaccinia virus vaccine, has been advised by the Advisory Committee on Immunization Practices (USA) for the vaccination of the laboratory personnel that routinely handle human infectious orthopoxviruses such as monkeypox, cowpox and variola viruses, and the medical personnel dealing with patients infected with vaccinia virus [11]. However, ACAM2000 has been reported to have side effects such as post-vaccinal encephalitis, eczema vaccinatum and progressive vaccinia [11].The use of ACAM2000 also poses safety concerns since it contains live vaccinia virus [11]. Further, many antivirals which have been employed for the treatment of orthopoxvirus, for instance, tecovirimat (SIGA Technologies, New York, NY, USA), inhibit viral DNA replication [12,13]. Tecovirimat is effective against monkeypox, variola, cowpox and vaccinia viruses in several animal models *viz.* ground squirrels, mice and cynomolgus monkeys [12,13]. As per the guidelines from the Center for Disease Control and Protection (CDC), USA, tecovirimat could be used for the treatment of monkeypox in an outbreak. Similarly, cidofir and vaccinia immune globulin (VIG), which are approved by the FDA for cytomegalo and vaccinia viruses, respectively, could also be used for monkeypox during an outbreak [14,15]. Apart from the drugs approved by the FDA, several approaches are being explored to intervene in the growth and outspread of MPXV, for instance, human interferon-βa is found to inhibit MPXV production and dissemination in mammalian cell lines [16]. Intriguingly, vaccinia virus LC16m8, which lacks the expression of the B5R membrane protein, protects against MPXV after immunization in cynomolgus monkeys [17]. Similarly, another attenuated vaccinia virus, NYCBH, after deletion of an immune evasion gene, is found to be effective in cynomolgus monkeys against monkeypox virus [18]. Nevertheless, there are no specific vaccines or antivirals available for the monkeypox infection in humans. The SARS-CoV-2 pandemic has already created the vigilance among scientific community to prepare beforehand for any highly contagious human viruses. Thus, it is of the utmost important to find novel strategies for developing potential therapies for monkeypox virus to prevent any such calamity in the future.

Interestingly, with the recent advances in the field of immunoinformatics, the pace of the vaccine development process has been relatively accelerated [19,20,21,22,23,24]. Based on immunoinformatics, we have identified different epitopes specifically for targeting multiple serotypes of the dengue virus [24]. Further extending this exciting computational-based vaccine design approach, our team has recently designed a vaccine for canine circovirus [24,25]. In the present study, we targeted different glycoproteins and membrane proteins (Table 1) of MPXV to identify immunogenic B-cell and CD4^+^ and CD8^+^ T-cell epitopes in order to design a potential vaccine for the monkeypox virus.

The final predicted vaccine is a multiepitope vaccine (MEV) construct that comprises all identified epitopes fused altogether to elicit an optimal immune response. Afterwards, the structural, immunogenic and physicochemical parameters were evaluated for the designed MPXV-MEV. Additionally, molecular docking was performed to investigate the affinity of the vaccine construct towards the human toll-like receptor 5 (TLR5). Later, the molecular dynamics (MD) simulations confirmed the stability of the MPXV-MEV construct with the TLR5 receptor and the associated interactions. Further, immune simulations were carried out to ascertain the immune response profile of the vaccine candidate.

## 2. Methodology

### 2.1. Retrieval and Analysis of Protein Sequence

The sequence of the MPXV strain W-Nigeria (Accession number: KJ642615.1) was obtained from the GenBank database. A total of 176 proteins in the proteome of MPXV strain W-Nigeria were characterized, where we filtered only glycoproteins and membrane proteins for further analysis, see Table 1. Further, the antigenicity and allergic potential of all the chosen proteins were determined. Vaxijen v2.0 webserver was used to determine the antigenicity [26]. Vaxijen v2.0 predicts the antigenic peptides with 70–89% accuracy and employs the alignment-free approach, which is based on the auto cross covariance (ACC) transformation of protein sequences into uniform vectors of principal amino acid properties. In the Vaxijen v2.0 webserver, the target organism selected was the virus and the protein sequences in plain format were used as input. AllergenFP v1.0 webserver was used to predict the allergic potential, since this server was found to be the most accurate in identifying both allergens and non-allergens in comparison with different tools such as AlgPred, AllerTOP, AllerHunter, and APPEL [27]. In the AllergenFP server, amino acid sequences were provided as plain text (single letter code).

### 2.2. Prediction of T-Cell and B-Cell Epitopes and to Determine Their Antigenicity, Ctoxicity, Allergenicity and Interferon-γ Activation Potential

To predict T-helper cell epitopes that might bind to MHC class II molecules, the NETMHC 2.3 website was used, whereas for the identification of T- cytotoxic cell epitopes that might bind to MHC class I molecules and elicit a cellular immune response, we used the NETMHC 4.0 website [28]. The NETMHC 2.3 webserver was preferred over other tools such as PickPocket, PRPPRED, MULTIPRED, ADT, and KISS since this webserver was found to be better in the earlier investigations where all these different tools for predicting peptides that bind to MHC were compared [29,30]. The protein sequences were pasted in FASTA format in both NETMHC 2.3 and NETMHC 4.0 webservers and inputs and a peptide length of 9 were selected. To predict CD4^+^ T cell epitopes, the HLA alleles selected were DRB1_0101, DRB1_0301, DRB1_0401, DRB1_0701, DRB1_0801, DRB1_0901, DRB1_1001, DRB1_1101, DRB1_1201, DRB1_1301, DRB1_1501, and DRB1_1602. Further, to predict CD8^+^ T cells, the HLA alleles selected were HLA-A0101, HLA A0201, HLA-A0301, HLA-A2402, HLA-A260, HLA-B0702, HLA-B0801, HLA-B2705, HLA-B3901, HLA-B4001, and HLA-B5801. Default parameters of the NETMHC 2.3 and NETMHC 4.0 webservers were used for the threshold for both the strong and weak binders. The IEDB B cell epitope prediction website’s Bepipred linear epitope prediction 2.0 approach was used to predict the multiple linear B cell epitopes [31]. The Vaxijen v2.0 webserver was used to determine the antigenicity of the predicted B-cell and T-cell epitopes [26]. The webservers AllergenFP, ToxinPred and IFNepitope were used to determine the antigenic, toxic, allergic and interferon-γ activation potential of the epitopes [27,32,33].

### 2.3. Anlysing Epitope Conservancy

The epitopes conservation was predicted in membrane proteins and glycoproteins of the three monkeypox virus strains, namely MPXV-WRAIR7-61, Sierra Leone, and COP-58, by employing the IEDB Epitope Conservancy Tool [34]. The sequences of epitopes and the proteins from which the epitopes were identified for each strain were pasted as input in FASTA format. The epitopes that were conserved were selected among different monkeypox virus strains in order to overcome the limitations due to antigenic shift or drift. The epitopes exhibiting 100% identity in sequences among the selected monkeypox strains were selected since they possessed less probability of any mutation [34].

### 2.4. Designing a Vaccine Construct and to Determine Its Physiochemical Properties

Both the B cell and T cell epitopes which fulfilled the selection criterion of properties, such as antigenic, non-toxic, non-allergic and interferon-γ activation, were linked together using the GSS linker. Two adjuvants—flagellin protein (*Salmonella typhimurium*) and RS09—were also linked to form the multiepitope vaccine construct using the GGS linker [35,36]. In order to improve the stability of the MEV construct, Pan HLA DR-binding epitope was also added [37]. The ProtParam online tool was used to determine the physiochemical properties such as hydrophilicity, isoelectric point, number of amino acids, number of negatively or positively charged residues, solubility, aliphatic index, extinction co-efficient, and half-life [38]. Further, the webservers Vaxijen 2.0 and AllergenFP were used to determine the antigenicity and allergic potential of the vaccine construct.

### 2.5. Prediction of Immune Response Profile of MEV

The C-IMMSIM webserver (https://kraken.iac.rm.cnr.it/C-IMMSIM/, accessed on 10 August 2022) is a position-specific scoring matrices-based machine learning approach and is used to investigate the immune response profile of a vaccine construct. This online webserver was used to predict the immunogenicity and immune behavior towards the candidate MPXV-MEV in the present study [39]. An interval of four weeks as the minimum duration between the first two doses of the vaccine injection has been advised; however, the interval can be extended to eight weeks or 3–6 months in some cases [40,41]. Thus, the immune behavior of MPXV-MEV vaccine was analyzed using three injections at an interval of four weeks [42,43]. Default parameters were kept except for the time. The time steps used were kept equivalent to four and eight weeks.

### 2.6. Prediction of Binding Affinity of MPXV-MEV with TLR5 Using Molecular Modeling and Docking

The Alphafoldv2.0 program was employed to predict the 3-D structures of both the MPXV-MEV construct and immunogenic TLR5 [44,45]. Alphafoldv2.0, ProCheck and ProSA web server were then used to validate the tertiary structures of MPXV-MEV and TLR5 [46]. The pLDDT scores were available with Alphafoldv2.0 and the Ramachandran plots and Z-scores were generated using the ProCheck and ProSA webservers [46].

The HADDOCK server [47] was used for docking MPXV-MEV to TLR5 using the default parameters [47]. LQRVRELAVQ and EILDISRNQL sequences were predicted as the potential binding regions in flagellin and human TLR5 [45]. Thus, during the docking experiments, these sequences were defined as the part of ‘Active Residues’ while running the HADDOCK program.

### 2.7. Molecular Dynamic Simulations of MPXV-MEV Complexed with TLR5

Afterwards, the GROMACS 2019 simulation program was used to perform the molecular dynamic (MD) simulations [48]. The complex of TLR5 and MPXV-MEV was put into a cubic box and solvated with TIP3P water molecules to create a solvent layer of 10 Å thick. The parameters of the proteins were then modeled with Amber ff99SB-ILDN [49]. Then, an appropriate number of K^+^ ions were added to neutralize the charge. Further, the Joung–Cheatham ion model was used to add extra K^+^Cl^−^ ions to create the bulk ionic strength, i.e., 0.15 M [50]. The simulation box had 228,291 water molecules, 615 K^+^ ions, and 612 Cl^−^ ions. The system had a total number of atoms of 701,665. The minimization of the system was carried out with 50,000 steps using the steepest descent method with 1000 kJ/mol nm^2^ position restraint on heavy atoms of the protein. Further, minimization was performed with no restraint on the protein. Later, equilibration of each system was performed in the phased manner. As the first step, a 100 ps NVT simulation was performed with restraint on protein heavy atoms. Secondly, a 100 ps NPT simulation with restraint on protein heavy atoms was carried out. Production simulations were performed while using the NPT ensemble for 100 ns. A temperature of 300 K was maintained with velocity rescaling with a 0.1 ps coupling time. Parrinello–Rahman barostat was used to maintain the pressure of 1 atm for NPT simulations with a coupling time of 2 ps [51]. Then, the leapfrog algorithm was used to integrate the equations of motion with a time step of 2.0 fs. The particle mesh Ewald (PME) summation was used to evaluate the total electrostatic interactions [52]. Coulomb and Van der Waals cut-offs of 1.0 nm were performed. In order to mimic the bulk behavior, the periodic boundary conditions in all directions were employed. The LINCS algorithm constrained the bond lengths with hydrogen [53]. At an interval of every 10 ps, the coordinates were kept collecting in the trajectory files. GROMACS tools were used for the trajectory processing and for most of the analysis. Three independent simulations, starting from different initial velocities and each for 100 ns, were performed. PYMOL and VMD softwares were used to create the molecular graphics images [54,55]. In-house Python scripts were used to plot the graphs. Indeed, our team has successfully used a similar modeling and MD simulation to investigate the structural stability of protein and nucleic acid systems [56,57,58,59,60].

## 3. Results

### 3.1. Protein Sequence Retrieval and Analysis

Out of 176 proteins, 18 proteins were identified. These proteins are classified as glycoproteins and membrane proteins in the proteome of the monkeypox virus strain W-Nigeria. Table 1 lists these proteins along with their predicted antigenic and allergic potential. Filtering those proteins possessing both antigenic and non-allergic potential resulted in the identification of 10 glycoproteins or membrane proteins, see Table 1.

### 3.2. Prediction of T Cell and B Cell Epitopes and Analysis of Their Antigenic, Allergic, Toxic and Interferon-γ Activation Potential

Strong binder epitopes of the MHC alleles, i.e., MHC I and MHC II, were first identified. The number of epitopes that were determined as strong binders for MHC I was 384 (see Table S1). Similarly, the number of epitopes identified as strong binders for MHC II was 256 (see Table S2). Further, 15 B cell epitopes were predicted (see Table S3). Epitopes with a Vaxijen antigen score ≥ to 1.0 along with other filtering parameters, such as antigenic, non-allergic, non-toxic properties and interferon-γ potential, were finally selected for the candidate monkeypox MEV design. Altogether, seven epitopes (two CD4^+^ T cell epitopes, four CD8^+^ T cell epitopes and one linear B cell epitope) fulfilled the selection criterion and were chosen for designing the final MPXV-MEV (Table 2). All the selected seven epitopes had 100% sequence identity in the MPXV-WRAIR7-61, Sierra Leone, and COP-58 strains of monkeypox virus. Further, the epitopes population coverage analysis was performed using the IEDB population coverage analysis tool, see Supplementary Information for more information. The MHC-II (CD4^+^ Tcell) epitopes, which are RIYFVSLSL and FSIGGVIHL and MHC-I (CD8^+^ Tcell) epitopes, which are IYFVSLSLL, LKHKYGCSL, AYTSISVVF and RYPIIDIKW, showed a world population coverage of 85.97% and 87.03% respectively.

### 3.3. Engineering Vaccine and to Determine Its Physiochemical Properties

**Figure 1:**
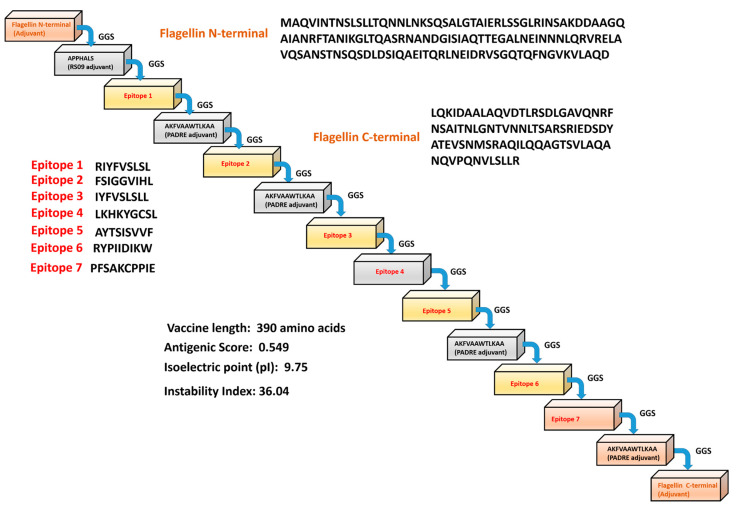
Scheme of the predicted monkeypox multi-epitope vaccine (MPXV-MEV) construct.

The epitopes identified were conjugated using adjuvants and Pan HLA DR-binding epitopes (PADRE) to develop the MEV construct. The final predicted vaccine construct has 390 residues (Figure 1). The MPXV-MEV construct was predicted as stable, antigenic and non-allergic in nature. The physiochemical properties of the MEV construct, other than the aforementioned, are provided in the Supplementary Information, see Table S4.

### 3.4. Performing Modeling and Docking of TLR5 MPXV-MEV Construct

**Figure 2:**
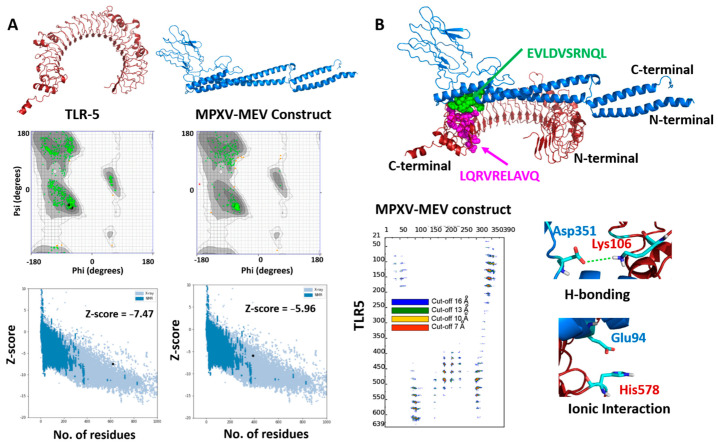
(**A**) Modeled 3D structures of (**A**) TLR5 and MPXY-MEV construct. Ramachandran plots and Z-scores calculated by Pro-SA webserver below the predicted structures. (**B**) Docked structure of MPXV-MEV and TLR5 as blue and red cartoons. The hotspot residues used for information-driven docking illustrated as spheres in green and magenta colors for the MEV construct and TLR5 receptor, respectively. Distance contact maps are also plotted, indicating the residues in contact between MPXV-MEV and the TLR5 receptor along with two representative molecular interactions between amino acid residues of TLR5 and the MPXV-MEV construct.

The 3D structures of both MPXV-MEV and the immunogenic TLR5 receptor were predicted using the Alphafoldv2.0 program [44,45]. Since no experimental structure is available for TLR5, the structure of TLR5 (Uniprot id: D1CS82) was obtained from AlphaFold prediction, which resulted in high pLDDT values with confidence scores >90% for most of the residues, indicating high confidence for prediction, see Figure S1. We particularly targeted the ectodomain residues (amino acids from 1–639) of the topological domain, which are primarily involved in the interaction with the extracellular signal; however, the transmembrane and TIR domain residues (amino acids from 640–836) are precluded from consideration in our analyses. Next, the structural prediction of the MPXV-MEV construct using Alphafold resulted in high pLDDT values with confidence scores > 90% for the N- and C-terminal regions of the vaccine construct. However, a low confidence score with pLDDT values of <50% were predicted for the regions where adjuvants/linkers and epitope sequences were present (amino acids from142–303), see Figure S1. Further, the quality of predicted structures was confirmed with a Ramachandran plot and the Z-scores of the modelledTLR5 and MPXV-MEV construct from the ProSA webserver [46]. For the MPXV-MEV construct, 96.7% of residues were observed in the favored regions; 2.9% residues were identified in the allowed and generously allowed regions; however, 0.4% residues were found under disallowed regions, see Figure 2A. Of the amino acids, 98.9% were observed in the core acceptable region; however, the other 1.1% were observed under the allowed region and generously allowed region for the TLR5 receptor, see Figure 2A. In order to investigate the interaction between the MEV construct and TLR5 involved in the immune response, molecular docking of MPXV-MEV was performed with TLR5 using the HADDOCK 2.4 web server and the default parameters [44]. Since flagellin, a bacterial adjuvant, specifically interacts with TLR5 which might invoke the innate immune response, an information-driven docking was performed, based on the information about specific interacting residues, to drive the docking simulations [36]. An analogous approach using a complementary hydropathy between the flagellin and TLR5 has previously been used for predicting the potential binding regions and the structure of the overall complex [61]. In a recent study, it has been shown that the potential binding regions identified for flagellin and human TLR5 were LQRVRELAVQ and EILDISRNQL [45]. Thus, during the docking experiments, these sequences were defined as the part of ‘Active Residues’ while running the HADDOCK program. The top ranked cluster containing the lowest HADDOCK score was selected as a final structure of the MPXV-MEV and TLR5 complex. The molecular docking between the MPXV-MEV construct and the TLR5 receptor is shown in Figure 2B. Further, we specifically computed the “distance range maps” for the docked complex using the COCOMAPS tool, see Figure 2B. In order to define a contact, a cut-off distance of 5 Å between two atoms was used. Forty-six contacts existed between hydrophilic residues, 50 were observed between hydrophilic and hydrophobic residues, and 11 existed in between two hydrophobic residues.

### 3.5. Structural Stability of the MPXV-MEV Complexed with TLR5

**Figure 3:**
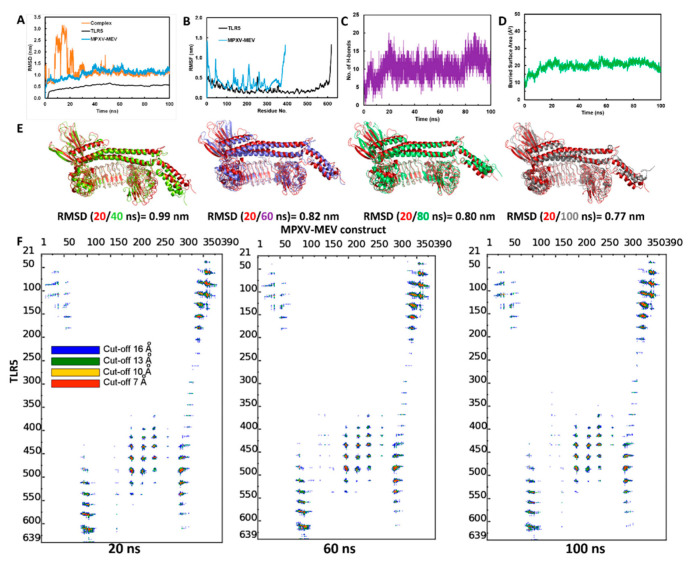
(**A**) Backbone RMSD plots of the docked complex and the individual chains of TLR5 and MPXV-MEV construct; (**B**) RMSF plots; (**C**) Hydrogen bonds analysis between TLR5 and MPXV-MEV during MD simulations; (**D**) Buried area of TLR5 and MPXV-MEV construct; (**E**) Superimposition of snapshots at every 20 ns of the TLR5 and MPXV-MEV constructs with their respective RMSD; (**F**) Contact maps showing inter-molecular contacts where the dots at the crossover of two amino acids have been colored in red, yellow, green and blue if any pair of atoms between two amino acids is closer than 7, 10, 13 and 16 Å.

MD simulations were performed to study the structural stability of the docked complex of MPXV-MEV and TLR5 [60]. GROMACS software was used to perform three different MD simulations, each 100 ns long, starting with different initial velocities [26], which produced very similar results, see Figure S2. Herein, we discuss the results for one of the simulations. To investigate the stability the MPXV-ME complexed with TLR5, the root mean square deviation (RMSD) of the Cα atoms from their initial position was monitored as a function of simulation time. It is evident from Figure 3A that the structure of the complex stabilizes after 30ns simulation time. The RMSD calculated for the docked TLR5 and MPXV-MEV complex after initial 30 ns of simulations is 1.08 ± 0.1 nm. Further, RMSD values were plotted separately for TLR5 and MPXV-MEV as well, see Figure 3A. Indeed, TLR5 alone was considerably stable after 30 ns (see Figure 3A and Figure S2A), with an average RMSD of 0.52 ± 0.04 nm. The predicted MPXV-MEV had flexibility with an RMSD value of 1.17 ± 0.09 nm. However, the RMSD values observed for trial-2 of MPXV-MEV are still not stable during the 100 ns simulation run, which may indicate a longer simulation run is required for attaining stability. Root mean square fluctuation (RMSF) was plotted separately for the TLR5 and the MPXV-MEV construct in order understand the structure’s flexibility on a residue basis. The elevated RMS fluctuations were observed for the highly flexible regions, see Figure 3B. It is evident that the Cα atoms of the entire TLR5 structure have a limited flexibility with an average RMSF of 0.22 ± 0.12 nm. On the other hand, a high fluctuation was found, particularly for the regions associated with N- and C-terminal of flagellin in the MPXV-MEV construct with an RMSF of 0.41 ± 0.20 nm and 0.38 ± 0.19 nm, respectively. Additionally, the number of intermolecular hydrogen bonds in the complex of TLR5 and MPXV-MEV remained constant after 30ns simulation time (Figure 3C). The buried surface area at the interface of the MPXV-MEV and TLR5 complex was stable throughout the simulation time, which clearly implies the stability of interface interaction between MPXV-MEV and TLR5 (Figure 3D). Further, we calculated the interaction energies between TLR5 and MPXV-MEV, i.e., the Lennard–Jones (E(LJ)) potential component, which stands for the Van der Waals interactions and the coulombic component (E(Coul)), representing the electrostatics interaction. From Figure S3, it is evident that both the electrostatic and LJ components contribute almost equally to the stabilization of the overall TLR5 and MPXV-MEV complex, with electrostatics energy of −714.65 ± 90.9 kcal/mol and an E(LJ) contribution of −665.89 ± 61.5 kcal/mol.

The superimposition of complex structures extracted every 20 ns during the simulation time resulted in good overlap, with RMSD values below 1 nm (Figure 3E); a limited flexibility was only observed in the terminal regions of the MPXV-MEV construct. An interface analysis was performed using the COCOMAPS tool for the same selected snapshots [62,63] (see, Figure 3F). Distance range maps were specifically plotted. The dots at the crossover of two residues belonging to the MPXV-MEV construct and to TLR5 are represented as red, yellow, green and blue if any pair of their atoms is closer than 7, 10, 13 and 16 Å. It is evident from Figure 3F that the interface remains stable for the selected snapshots in terms of inter-residue contacts, despite the observed peripheral flexibility of the MPXV-MEV construct.

**Figure 4:**
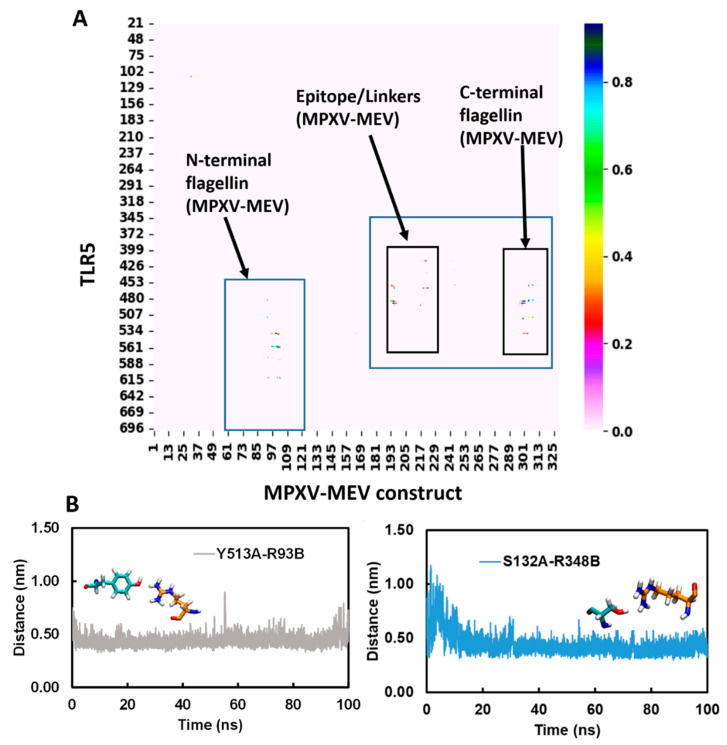
(**A**) Consensus map of 1000 MD snapshots obtained from MDcons tool. The flagellin N- and C-terminal and the region of epitope/linkers on MPXV-MEV interacting with TLR5 are highlighted in blue boxes; (**B**) Time evolution of conserved contacts (dark blue dots as in **A**) between TLR5 and the MPXV-MEV.

Further, the MDcons program was used to assess the stability of the complex between MPXV-MEV and TLR5, and the conservation of inter-residue contacts (ICs) at the interface along the simulation time was plotted [64,65,66,67,68,69]. The similarity between different snapshots focused on the region of interest, which is the biomolecular interface, could be efficiently measured by the conservation of ICs under dynamic conditions between macromolecules giving a stable complex. Figure 4A,B illustrate a consensus map of 1000 MD snapshots and distances along the simulation time for two selected conserved ICs, respectively. The overall conservation of the ICs at the MPXV-MEV and TLR5 interface during the MD simulations is illustrated in the MD consensus map which clearly indicates that many contacts between the MPXV-MEV construct and TLR5 remained stable throughout the simulation time (Figure 4A). MDcons analysis produced C_50_ and C_70_ values of 0.87 and 0.51, implying that 87% and 51% of ICs were kept conserved for at least 50% and 70% of the frames. Hence, quite a high conservation of the complex interface during the simulation time was observed. The ICs remained stable over the simulation time and two such examples are shown in Figure 4B. Notably, the H-bond existing between Tyr513 from TLR5 and Arg93 from the N-terminal of flagellin on the MPXV-MEV construct remained stable throughout the simulation time with an average Tyr513(OH)-Arg230(CZ) distance of 0.41 ± 0.05 nm. The hydrogen bonding interaction between Ser132 of TLR5 and Arg348 from the C-terminal of flagellin on the MPXV-MEV construct was also observed to remain stable with an average Ser132(OG)-Arg348(CZ) distance of 0.44 ± 0.05 nm.

### 3.6. Immune Simulation

**Figure 5:**
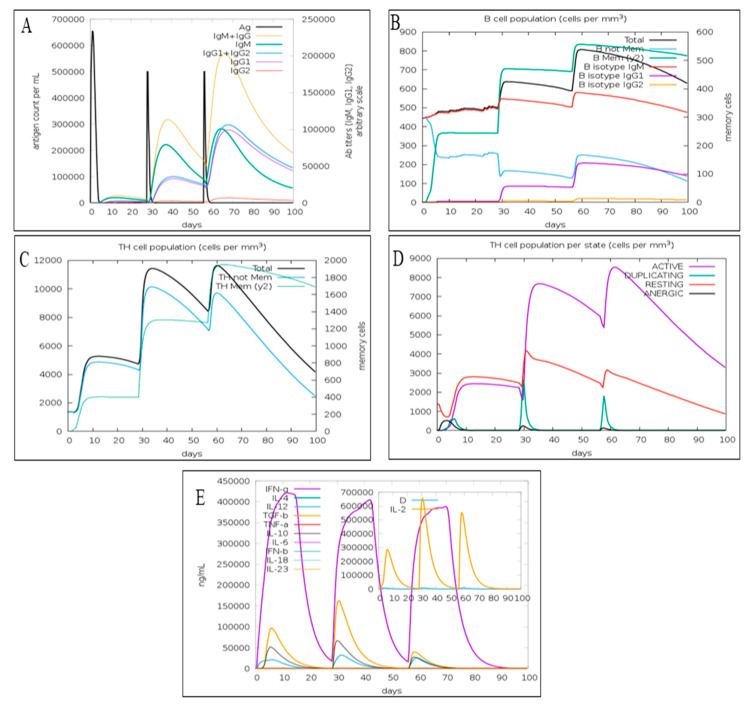
The immune response predicted using C-IMMSIM (**A**) antigen count and antibody titer with specific subclass; (**B**) B cell; (**C**) CD4^+^ T cell; (**D**) CD4^+^ T cell population per state; (**E**) cytokines and interleukins.

The immune simulation demonstrated that the first exposure to the candidate monkeypox MEV did not elevate the antibody titer significantly. However, the next two injections considerably increased the antibody titer (IgM + IgG, IgM, IgG1 + IgG2, and IgG1) relative to the first dose of MEV (Figure 5A). The third dose also elevated IgG2 titer. The C-IMMSIM also determined that candidate monkeypox MEV has the potential to grow the total B-lymphocytes population after each injection (Figure 5B). An increase in B isotype IgG1 and B isotype IgM were also predicted (Figure 1B). Further, the expression of B memory cells was increased after each vaccine dose, implying a robust secondary immune response activation (Figure 5B). The total population of CD4^+^ T l was also increased following every vaccination. Moreover, the population of memory T cells was increased following each vaccination (Figure 5C). However, the total CD4^+^ T cell population remained the same after the second and third doses of MEV (Figure 5C). Furthermore, there was increase in the population of active CD4^+^ T cells after the vaccination (Figure 5D). The resting and duplicating CD4^+^ T cells were increased after the first two injections; however, after the third injection, their population slightly decreased (Figure 5D). Thus, a rise in memory B cells and resting T cells after vaccination implies the activation of the adaptive immune response on which the vaccination is based. The candidate MPXV-MEV vaccination also stimulated the production of interleukin-10, interleukin-12, interferon-γ and TGF-β (Figure 5E). Initially, an increase in these cytokines and interleukins was observed after the first two vaccinations; however, their concentration was decreased after the third dose compared to the first two shots (Figure 5E). Overall, the immune simulations predicted that candidate MPXV-MEV vaccination could activate the immune response.

## 4. Discussion

Monkeypox is an orthopoxvirus, which is responsible for infectious diseases such as cowpox, smallpox, camelpox, and horsepox [70]. The monkeypox contains double-stranded DNA which is approximately 197 kb and contains 190 non-overlapping open reading frames [71]. Monkeypox virus has been detected in various animals such as Gambian rats, Asian monkeys, rhesus macaques, prairie dogs and rope squirrels [72,73]. Moreover, Gambian-pouched rats have also been speculated to be the reservoir host species of MPXV [74]. Monkeypox is reported to infect a broad range of hosts; intriguingly, the natural host of the virus has still not been identified [73]. Either of the two modes of transmission, i.e., animal-to-human or human-to-human, can infect humans [73]. The symptoms include skin rashes, fever, fatigue, lesions, and lymphadenopathy [75]. Further complications include dehydration, vomiting, conjunctivitis, encephalitis, pharyngitis, tonsillitis, and diarrhea [75].

The currently available vaccines that have provided immunization against monkeypox include FDA approved ACAM2000 or NYCBH and LC16m8 which are under clinical trials. These vaccines are live, attenuated vaccinia viruses which face challenges of safety and efficacy in chronic infections [76,77]. Furthermore, there is a risk of virulence reversion due to complementing mutations in vaccine recipients or production hosts during vaccine viruses’ replication [76,77]. Moreover, in light of the recent multi-country monkeypox outbreak and the lack of any specific treatment for the same, it has become imperative to look for new ways to treat and protect the human population from monkeypox. Hence, an immunoinfromatics approach seems to be exciting for designing a potential vaccine candidate to explore an alternative to the live attenuated virus. Furthermore, the epitope-based vaccines could help to overcome challenges such as genetic variations, antigenic shift and antigenic drift. Previously, various epitope-based vaccine candidates against pathogens such as bacteria, viruses, fungi, parasites and cancers have been designed using an in silico immunoinformatics approach [23,42,78,79,80,81,82,83,84,85,86,87,88]. Recently, an MEV showing a T-cell response for Q fever in cynomolgus macaques has been reported [79]. Quite recently, immunoinformatics-based designs of vaccines have also been proposed, targeting various proteins for MPXV [89,90,91,92].

The epitopes ISPDGCYSL and LTFDYVVTF, which were derived from the F8L protein of the monkeypox virus, have been reported to activate the CD8^+^ T cell population and the release of interferon-γ in rhesus macaques [93]. Interestingly, an epitope-based vaccine, VennVax, exhibits 100% protective efficacy against vaccinia virus by activating T cell response in the humanized mouse model. Interestingly, VennVax has recently been found to be effective as an MPXV cure as well [94,95].

In the present study, the membrane proteins and glycoproteins were targeted for predicting antigenic epitopes to design a potential MEV candidate against monkeypox. These proteins are major constituents of pathogenic viruses and play a significant role in immunity and pathogenesis [96], and are involved in the attachment to the host cellular receptors and virus–host membrane fusion [96]. In this study, seven epitopes (two cell epitopes, four cell epitopes and one linear B cell epitope) possessed antigenicity, non-allergenicity, and non-toxicity. Furthermore, these epitopes were also predicted to activate interferon-γ production. Interferon-γ modulates both the innate and adaptive immunity while contributing to the antiviral defense system [97]. Then, the selected epitopes were linked with adjuvants and the PADRE sequence to design a novel monkeypox MEV candidate. Furthermore, the candidate monkeypox MEV was predicted to be antigenic and non-allergic. Further, the docking and MD simulations of MPXV-MEV complexed with the TLR5 receptor resulted in a stable interaction pattern especially at the biological interface of this complex. The computational analyses emphasize that the novel MPXV-MEV construct identified is a potent immunogen which exhibits both non-allergenicity and non-toxicity. Interestingly, the vaccine construct is dissimilar to human proteins (taxid: 9606; *Homo sapiens*) in a protein–protein BLAST analysis, indicating that it could be safely used for humans.

## 5. Conclusions

The immunogenic and non-allergic glycoproteins and membrane proteins for MPXV have been targeted in order to predict epitopes for T-cells and B-cells that could be used to design a candidate MEV against MPXV. The predicted MPXV-MEV is antigenic and non-allergic in nature and interacts strongly with human TLR5. In silico immune simulation of the MEV construct predicted that the vaccine candidate could elicit an immune response in humans.

## Figures and Tables

**Table 1:** Glycoproteins and membrane proteins of monkeypox virus strain W-Nigeria and their antigenic and allergic potential.

GenBank Protein ID	Protein Name	Length (Amino Acids)	Vaxijen Score	Allergen (AllergenFP)
AIE40790.1	putative membrane-associated glycoprotein	1880	0.5262 (Antigenic)	Allergen
AIE40786.1	IFN-alpha/beta-receptor-like secreted glycoprotein	352	0.5453 (Antigenic)	Non-allergen
AIE40780.1	bifunctional 21 kDa precursor protein of 18 kDa membrane protein	182	0.4395 (Antigenic)	Allergen
AIE40778.1	EEV type-I membrane glycoprotein	317	0.5786 (Antigenic)	Non-allergen
AIE40774.1	bifunctional hemagglutinin/type-I membrane glycoprotein	313	0.4638 (Antigenic)	Allergen
AIE40766.1	putative type-I membrane glycoprotein	196	0.5230 (Antigenic)	Non-allergen
AIE40764.1	bifunctional secreted glycoprotein	221	0.3864 (Non-antigenic)	Allergen
AIE40763.1	CD47-like putative membrane protein	277	0.4324 (Antigenic)	Non-allergen
AIE40759.1	EEV glycoprotein	168	0.3728 (Non-antigenic)	Non-allergen
AIE40758.1	bifunctional EEV membrane phosphoglycoprotein	181	0.4998 (Antigenic)	Allergen
AIE40739.1	IV and IMV membrane protein	53	0.7480 (Antigenic)	Non-allergen
AIE40738.1	phosphorylated IMV membrane protein	90	0.4759 (Antigenic)	Non-allergen
AIE40737.1	IMV membrane protein	70	0.5019 (Antigenic)	Non-allergen
AIE40733.1	IMV membrane protein	100	0.3923 (Non-antigenic)	Non-allergen
AIE40718.1	IMV membrane protein	304	0.5316 (Antigenic)	Non-allergen
AIE40702.1	late 16 kDa putative membrane protein	133	0.7559 (Antigenic)	Non-allergen
AIE40669.1	membrane protein	273	0.4199 (Antigenic)	Non-allergen
AIE40657.1	palmytilated EEV membrane protein	372	0.4754 (Antigenic)	Allergen

**Table 2:** The epitopes identified for vaccine engineering along with the analysis of their toxic, allergic, antigenic, and interferon-γ activation potential.

Epitope	Peptide or Protein	Vaxijen	Antigenicity	Allergenicity	Toxicity	Interferon Activation
CD4^+^ T cell	RIYFVSLSL/AIE40786.1	1.6615	Yes	No	No	Yes
CD4^+^ T cell	FSIGGVIHL/AIE40778.1	1.2283	Yes	No	No	Yes
CD8^+^ T cell	IYFVSLSLL/AIE40786.1	1.4551	Yes	No	No	Yes
CD8^+^ T cell	LKHKYGCSL/AIE40766.1	1.2793	Yes	No	No	Yes
CD8^+^ T cell	AYTSISVVF/AIE40763.1	1.1050	Yes	No	No	Yes
CD8^+^ T cell	RYPIIDIKW/AIE40702.1	2.5629	Yes	No	No	Yes
B cell	PFSAKCPPIE/AIE40786.1	1.1314	Yes	No	No	Yes

## Data Availability

The data presented in this study are available in the article and the Supplementary Material.

## Supplementary Materials

The following supporting information can be downloaded at: https://www.mdpi.com/article/10.3390/v14112374/s1, Figure S1: Modeling of MPXV-MEV construct and TLR5 receptor using AlphaFold; Figure S2: Molecular dynamics simulations results obtained from three independent simulations for Root Mean Square Deviation (RMSD) for (A) TLR5 and (B) MAXV-MEV. (C) Total number of hydrogen bonds between TLR5 and MPXV-MEV; Figure S3: Interaction energy between TLR5 and MAXV-MEV obtained from three independent simulations; Table S1: Prediction of helper T cell epitopes and their antigenicity, allergenicity, toxicity and interferon-γ inducing ability; Table S2: Prediction of cytotoxic T cell epitopes and their antigenicity, allergenicity, toxicity and interferon-γ inducing ability; Table S3: Prediction of B cell epitopes and their antigenicity, allergenicity, toxicity and interferon-γ inducing ability; Table S4: Physiochemical Properties of the MPXV-MEV construct.
