# Supplementary material for: Immunoinformatics-Aided Design of a Peptide Based Multiepitope Vaccine Targeting Glycoproteins and Membrane Proteins against Monkeypox Virus
Source: Viruses. 2022 Oct 27;14(11):2374. doi: 10.3390/v14112374 (PMC9693848; doi:10.3390/v14112374)
Supplement: Supplementary file 1 [file viruses-14-02374-s001.zip › viruses-1976280-supplementary.pdf]

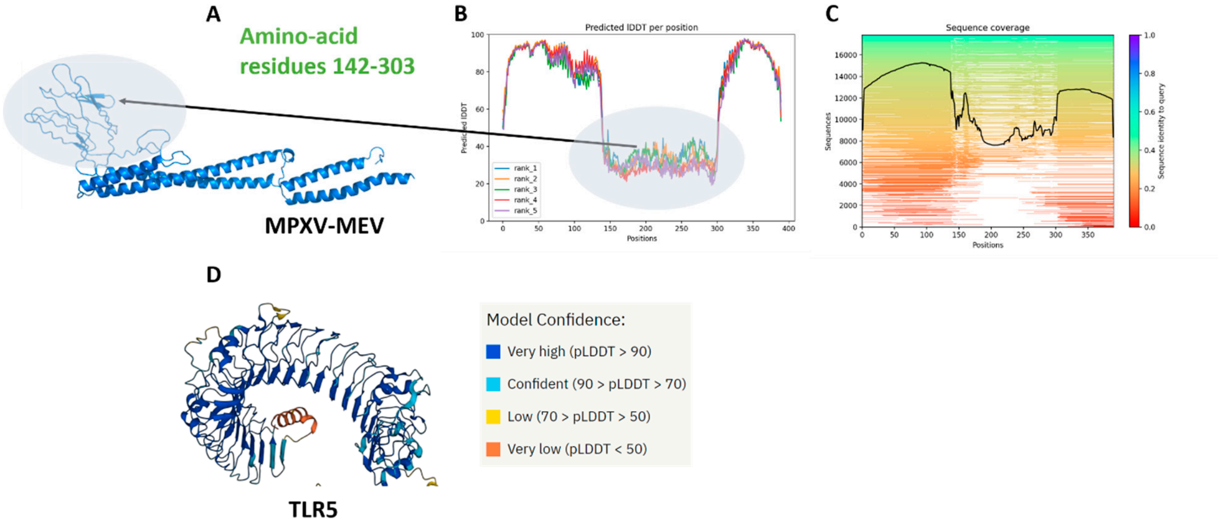

Figure S1: Modeling of MPXV-MEV construct and TLR5 receptor using AlphaFold. A) Modeled structure of MPXV-MEV with residues 142-303 (where adjuvants, epitopes and linkers are inserted) in grey highlighted area; B) Predicted LDDT values for positions in MPXV-MEV construct for different models obtained from AlphaFold prediction; C) Sequence coverage map of MPXV-MEV vaccine construct; D) Modeled structure of TLR5 receptor with the predicted LDDT values.

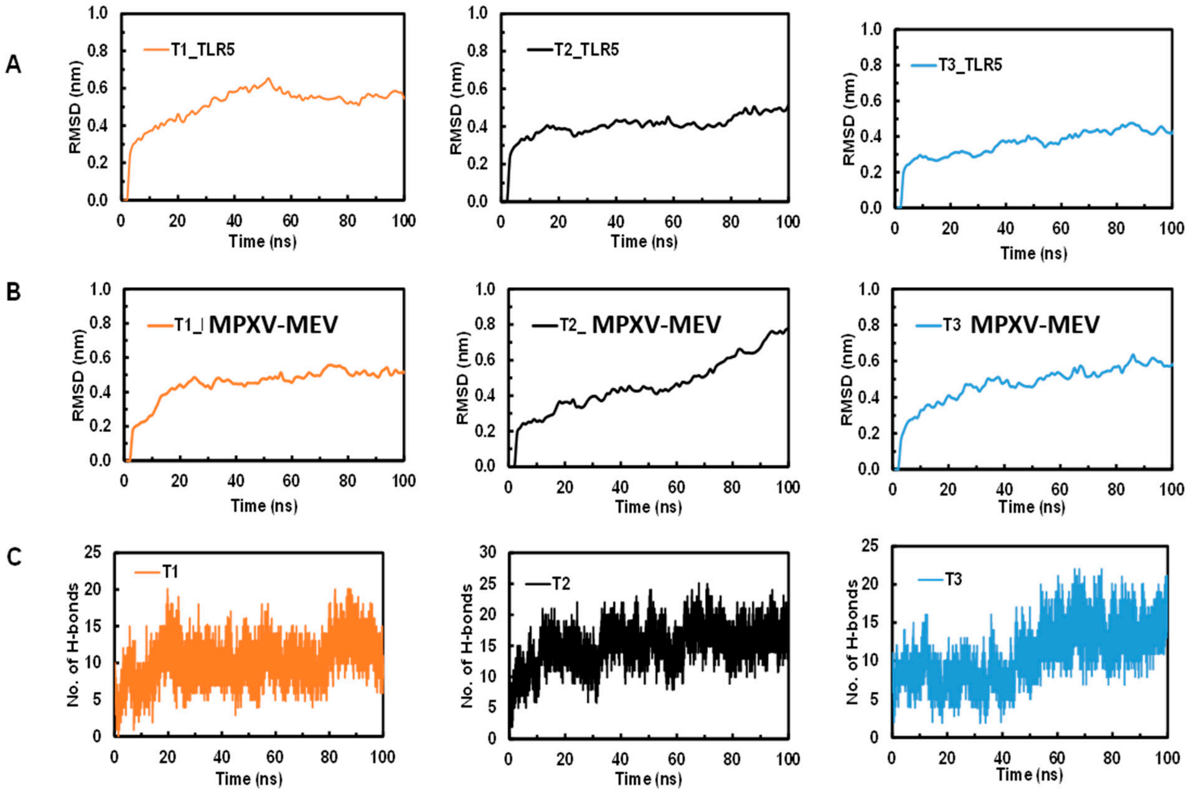

Figure S2: Molecular dynamics simulations results obtained from three independent simulations for Root Mean Square Deviation (RMSD) for A) TLR5 and B) MAXV-MEV. C) Total number of hydrogen bonds between TLR5 and MPXV-MEV.

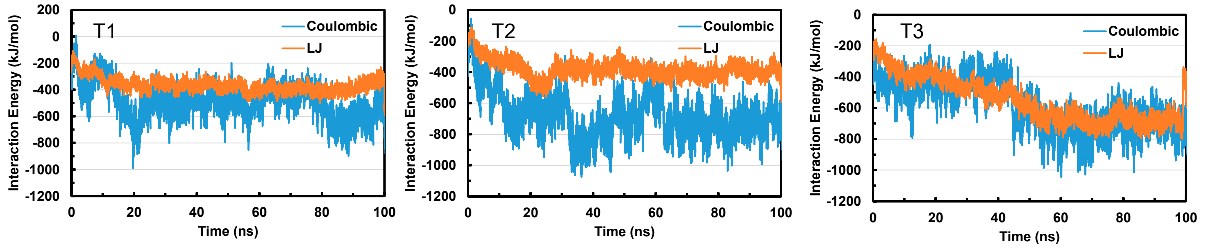

Figure S3: Interaction energy between TLR5 and MAXV-MEV obtained from three independent simulations. Lennard Jones (LJ) and Coulombic interaction energy is shown with orange and blue lines, respectively.

Method for calculating the population coverage:

Default values of the “number of epitopes” and “query by” box were chosen. “World” was chosen for “select area(s) and/or population(s)”. For epitopes RIYFVSLSL and FSIGGVIHL “class II separate” option and for epitopes IYFVSLSLL, LKHKYGCSL, AYTSISVVF and RYPIIDIKW ”class I separate” option in “select calculation options” were used. Then the epitopes were pasted in the “epitope box”. For MHCII epitopes DRB1_0101, DRB1_0301, DRB1_0401, DRB1_0701, DRB1_0801, DRB1_0901, DRB1_1001, DRB1_1101, DRB1_1201, DRB1_1301, DRB1_1501, and DRB1_1602 alleles were selected and for MHC-1 epitopes HLA-A0101, HLA A0201, HLA-A0301, HLA-A2402, HLA-A260, HLA-B0702, HLA-B0801, HLA-B2705, HLA-B3901, HLA-B4001, and HLA-B5801 alleles were chosen.

Table S1: Prediction of helper T cell epitopes and their antigenicity, allergenicity, toxicity and interferon-γ inducing ability

Protein ID	Allele	Peptide	Binding Affinity (nM)	Vaxijen score	Antigen/Non-antigen	Allergenicity	Toxicity	Interferon-γ inducing ability
AIE40786.1	DRB1_0101	FKLILDPKI	50.5	0.5910	Antigen	Allergen	Non-toxin	No
		YFVSLSLLL	1064.0	0.9237	Antigen	Allergen	Non-toxin	Yes
		YSILTSRGG	1346.5	0.8118	Antigen	Allergen	Non-toxin	No
	DRB1_0301	LFVDDVLIE	1385.5	-0.2442	Non-antigen	Allergen	Non-toxin	No
		VHYDDVRIK	1397.9	1.3493	Antigen	Non-allergen	Non-toxin	No
		IKNDIVVSR	1468.3	0.2927	Non-antigen	Non-allergen	Non-toxin	No
		LILDPKINV	2341.9	0.7445	Antigen	Allergen	Non-toxin	No
		INIDDFKYS	3227.5	0.8011	Antigen	Non-allergen	Non-toxin	No
		RIKNDIVVS	5468.8	0.4547	Antigen	Non-allergen	Non-toxin	No
		LIIHNPELE	7117.3	0.1541	Non-antigen	Allergen	Non-toxin	Yes
		IGLDFGVYS	8877.2	1.2209	Antigen	Allergen	Non-toxin	No
		KLILDPKIN	9353.2	0.6287	Antigen	Allergen	Non-toxin	No
	DRB1_0401	YFVSLSLLL	2452.3	0.9237	Antigen	Allergen	Non-toxin	Yes
		YYFDKTLTT	2987.7	0.2380	Non-antigen	Non-allergen	Non-toxin	No
		YLCTVTTKN	3891.7	1.6441	Antigen	Allergen	Non-toxin	No
		YAKHYNNIT	4585.9	0.6238	Antigen	Non-allergen	Non-toxin	No
		VYSILTSRG	6768.0	0.6613	Antigen	Non-allergen	Non-toxin	No
	DRB1_0701	FKLILDPKI	94.9	0.5910	Antigen	Allergen	Non-toxin	No
		LFHSYAIDI	108.8	0.4868	Antigen	Allergen	Non-toxin	No
		YFVSLSLLL	121.3	0.9237	Antigen	Allergen	Non-toxin	Yes
		LLLFHSYAI	131.4	0.2825	Non-antigen	Allergen	Non-toxin	Yes
		FDKTLTTTV	193.5	0.1539	Non-antigen	Non-allergen	Non-toxin	No
		KTLTTTVVL	261.3	0.1327	Non-antigen	Allergen	Non-toxin	No
		SAVSTSLFV	287.2	0.0182	Non-antigen	Non-allergen	Non-toxin	No

		IVVSRCKIL	319.5	0.1110	Non-antigen	Allergen	Non-toxin	No
	DRB1_0801	MKMKMMVRI	333.6	0.8614	Antigen	Allergen	Non-toxin	Yes
		MKMMVRIYF	445.0	0.3443	Non-antigen	Allergen	Non-toxin	No
		LLFHSYAID	630.9	0.3597	Non-antigen	Non-allergen	Non-toxin	No
		FKYSQAGKE	730.4	0.9583	Antigen	Allergen	Non-toxin	No
		GILYAKHYN	805.1	1.2526	Antigen	Allergen	Non-toxin	No
		YSILTSRGG	826.1	0.8118	Antigen	Allergen	Non-toxin	No
		LWIANYTSK	834.2	0.3380	Non-antigen	Allergen	Non-toxin	No
	DRB1_0901	FKYSQAGKE	335.8	0.9583	Antigen	Allergen	Non-toxin	No
		LFHSYAIDI	480.9	0.4868	Antigen	Allergen	Non-toxin	No
		YFVSLSLLL	877.1	0.9237	Antigen	Allergen	Non-toxin	Yes
		FKLILDPKI	976.4	0.5910	Antigen	Allergen	Non-toxin	No
		VIPSQDHRF	1294.5	1.0217	Antigen	Allergen	Non-toxin	Yes
	DRB1_1001	YFVSLSLLL	342.4	0.9237	Antigen	Allergen	Non-toxin	Yes
		YLCTVTTKN	835.4	1.6441	Antigen	Allergen	Non-toxin	No
		FKLILDPKI	976.6	0.5910	Antigen	Allergen	Non-toxin	No
	DRB1_1101	WERLEKNRR	805.0	-0.3554	Non-antigen	Non-allergen	Non-toxin	Yes
		YSILTSRGG	1834.1	0.8118	Antigen	Allergen	Non-toxin	No
		KMKMMVRIY	2424.7	0.2450	Non-antigen	Allergen	Non-toxin	Yes
		MKMKMMVRI	3912.1	0.8614	Antigen	Allergen	Non-toxin	Yes
	DRB1_1201	KMKMMVRIY	544.6	0.2450	Non-antigen	Allergen	Non-toxin	Yes
		CGILYAKHY	666.9	0.6483	Antigen	Allergen	Non-toxin	No
		IIGLDFGVY	820.9	0.9216	Antigen	Allergen	Non-toxin	No

		LSLLLFHSY	937.7	0.4192	Antigen	Non-allergen	Non-toxin	No
	DRB1_1301	MMKMKMMVR	5.4	0.6078	Antigen	Allergen	Non-toxin	No
		RRRQVSNKR	9.7	0.3556	Non-antigen	Non-allergen	Non-toxin	No
		MKMMVRIYF	15.1	0.3443	Non-antigen	Allergen	Non-toxin	Yes
		MKMKMMVRI	16.9	0.8614	Antigen	Allergen	Non-toxin	Yes
		ERLEKNRRR	35.1	-0.5015	Non-antigen	Non-allergen	Non-toxin	Yes
		WERLEKNRR	42.3	-0.3554	Non-antigen	Non-allergen	Non-toxin	Yes
		LEKNRRRQV	46.3	0.4224	Antigen	Non-allergen	Non-toxin	Yes
		RLEKNRRRQ	54.5	-0.4682	Non-antigen	Allergen	Non-toxin	Yes
		KMKMMVRIY	57.9	0.2450	Non-antigen	Allergen	Non-toxin	Yes
		TSKFSNRRY	58.3	0.4125	Antigen	Allergen	Non-toxin	No
		YTSKFSNRR	59.5	0.4382	Antigen	Non-allergen	Non-toxin	No
		VQGVVRSHV	65.6	-0.0028	Non-antigen	Allergen	Non-toxin	No
		SKFSNRRYL	67.0	0.1667	Non-antigen	Allergen	Non-toxin	Np
	DRB1_1501	LLLFHSYAI	224.0	0.2825	Non-antigen	Allergen	Non-toxin	Yes
		RIYFVSLSL	342.0	1.6615	Antigen	Non-allergen	Non-toxin	Yes
		FKLILDPKI	1876.8	0.5910	Antigen	Allergen	Non-toxin	No
		VRIYFVSLS	3942.9	1.4782	Antigen	Allergen	Non-toxin	No
	DRB1_1602	YFVSLSLLL	1861.3	0.9237	Antigen	Allergen	Non-toxin	Yes
		FKLILDPKI	3178.2	0.5910	Antigen	Allergen	Non-toxin	No
		LLLFHSYAI	3736.8	0.2825	Non-antigen	Allergen	Non-toxin	Yes
		RIYFVSLSL	3935.6	1.6615	Antigen	Non-allergen	Non-toxin	Yes
		ELIIHNPEL	5084.0	-0.1336	Non-antigen	Non-allergen	Non-toxin	No

AIE40778.1	DRB1_0101	WNPILPTCV	458.9	0.1552	Non-antigen	Allergen	Non-toxin	No
		YHIIIMALT	548.5	0.7969	Antigen	Non-allergen	Non-toxin	No
		YHSLDPNAV	1380.2	1.0653	Antigen	Allergen	Non-toxin	Yes
	DRB1_0301	VCETDKWKY	8635.7	-0.4923	Non-antigen	Non-allergen		
	DRB1_0401	FTLTGSPSS	5003.0	0.8750	Antigen	Non-allergen	Non-toxin	No
		VYSTCTVPT	6000.5	0.1518	Non-antigen	Allergen		
		YISCTANSW	6756.7	0.7152	Antigen	Allergen	Non-toxin	Yes
		LYEVNSTMT	7614.2	0.8315	Antigen	Non-allergen	Non-toxin	Yes
	DRB1_0701	YEVIGVSYI	196.4	1.1025	Antigen	Allergen	Non-toxin	No
	DRB1_0801	IMALTIMGV	585.9	0.8565	Antigen	Allergen	Non-toxin	Yes
	DRB1_0901	FSIGGVIHL	206.0	1.2283	Antigen	Non-allergen	Non-toxin	Yes
		IMALTIMGV	885.6	0.8565	Antigen	Allergen	Non-toxin	Yes
	DRB1_1001	YISCTANSW	1276.8	0.7152	Antigen	Allergen	Non-toxin	Yes
		YEVIGVSYI	1662.4	1.1025	Antigen	Allergen	Non-toxin	No
	DRB1_1201	LCVLPAVVY	1075.9	0.6499	Antigen	Non-allergen	Non-toxin	Yes
	DRB1_1301	IMALTIMGV	30.5	0.8565	Antigen	Allergen	Non-toxin	Yes
		IIIMALTIM	44.1	0.6886	Antigen	Allergen	Non-toxin	No
		LTIMGVIFL	55.2	0.5872	Antigen	Allergen	Non-toxin	No
		MKTISVVTL	67.4	0.8900	Antigen	Allergen	Non-toxin	No
	DRB1_1501	HIIIMALTI	2286.9	0.7663	Antigen	Non-allergen	Non-toxin	No
	DRB1_1602	FTLTGSPSS	4581.0	0.8750	Antigen	Non-allergen	Non-toxin	No
AIE40766.1	DRB1_0101	ICMLIASTL	481.7	0.7125	Antigen	Allergen	Non-toxin	No
		IVSLSGNLK	560.9	0.6211	Antigen	Non-allergen	Non-toxin	No
		FDRIYNNVV	1045.6	-0.6790	Non-antigen			

		CIAIIGYDS	1570.7	0.6721	Antigen	Allergen	Non-toxin	No
	DRB1_0301	ILTSKHKTY	6165.6	1.1168	Antigen	Allergen	Non-toxin	No
		LTYSSSIFR	8900.1	-0.1911	Non-antigen			
		DELDDNVFF	8991.8	-0.0805	Non-antigen			
		IIYVFKKIK	9835.0	0.5630	Antigen	Allergen	Non-toxin	Yes
		IYVFKKIKM	1025.7	0.3446	Non-antigen			
	DRB1_0401	WYKYINDRY	4846.6	-0.5812	Non-antigen			
		FDRIYNNVV	5448.2	-0.6790	Non-antigen			
		IYNNVVNIK	6360.4	0.4886	Antigen	Allergen	Non-toxin	Yes
		IISILTMSI	6494.5	0.5157	Antigen	Non-allergen	Non-toxin	Yes
		IMPVLTYSS	6778.5	0.4387	Antigen	Allergen	Non-toxin	No
		YRSTCIAII	7432.5	1.6925	Antigen	Non-allergen	Non-toxin	No
		IVSLSGNLK	7482.6	0.6211	Antigen	Non-allergen	Non-toxin	No
	DRB1_0701	YSLYRSTCI	22.6	0.5128	Antigen	Allergen	Non-toxin	No
		CMLIASTLI	25.9	0.5512	Antigen	Allergen	Non-toxin	No
		YRSTCIAII	56.0	1.6925	Antigen	Non-allergen	Non-toxin	No
		IFRFHSEDI	57.7	0.2452	Non-antigen			
		LIVSLSGNL	67.2	0.0008	Non-antigen			
		CSLNPSLIV	100.3	0.7126	Antigen	Allergen	Non-toxin	No
		LATSTSTIL	161.9	0.0871	Non-antigen			
		CSVNVSCLI	205.9	0.8331	Antigen	Allergen	Non-toxin	No
		FDRIYNNVV	236.9	-0.6790	Non-antigen			
	DRB1_0801	YFLKHKYGC	190.0	0.9444	Antigen	Allergen	Non-toxin	Yes
		IYVFKKIKM	235.0	0.3446	Non-antigen			
		IIYVFKKIK	291.2	0.5630	Antigen	Allergen	Non-toxin	Yes
		YVFKKIKMN	359.4	0.2841	Non-antigen			
		ILTMSIMPV	389.8	0.9840	Antigen	Non-allergen	Non-toxin	Yes
		TILTSKHKT	576.0	0.9669	Antigen	Allergen	Non-toxin	No

	DRB1_0901	YRSTCIAII	115.1	1.6925	Antigen	Non-allergen	Non-toxin	No
		LIVSLSGNL	174.4	0.0008	Non-antigen			
		ICMLIASTL	290.3	0.7125	Antigen	Allergen	Non-toxin	No
		LTMSIMPVL	299.6	1.1100	Antigen	Non-allergen	Non-toxin	No
		ILTMSIMPV	488.9	0.9840	Antigen	Non-allergen	Non-toxin	Yes
		LYRSTCIAI	566.4	1.1388	Antigen	Allergen	Non-toxin	No
		IFRFHSEDI	593.1	0.2452	Non-antigen			
		WYKYINDRY	1091.7	-0.5812	Non-antigen			
	DRB1_1001	YRSTCIAII	1439.2	1.6925	Antigen	Non-allergen	Non-toxin	No
		YKYINDRYN	1486.8	0.0098	Non-antigen			
	DRB1_1101	VFKKIKMNS	1070.1	0.1505	Non-antigen			
		IIYVFKKIK	1392.5	0.5630	Antigen	Allergen	Non-toxin	Yes
		YFLKHKYGC	1962.6	0.9444	Antigen	Allergen	Non-toxin	Yes
		YRYNFINRT	2001.1	0.0471	Non-antigen			
		TILTSKHKT	2164.8	0.9669	Antigen	Allergen	Non-toxin	No
		YVFKKIKMN	2387.1	0.2841	Non-antigen			
		IYVFKKIKM	2667.7	0.3446	Non-antigen			
		GYFLKHKYG	3265.8	0.3599	Non-antigen			
		YNFINRTFS	4297.5	0.5352	Antigen	Non-allergen	Non-toxin	No
	DRB1_1201	IYVFKKIKM	787.0	0.3446	Non-antigen			
		VSLSGNLKY	1509.0	1.0750	Antigen	Non-allergen	Non-toxin	No
		ILTSKHKTY	1515.3	1.1168	Antigen	Allergen	Non-toxin	No
		MSIMPVLTY	1521.8	0.8206	Antigen	Allergen	Non-toxin	No
		IELCYGNLY	1786.8	0.4470	Antigen	Non-allergen	Non-toxin	Yes
	DRB1_1301	IYVFKKIKM	25.9	0.3446	Non-antigen			
		TILTSKHKT	29.8	0.9669	Antigen	Allergen	Non-toxin	No
		MMKWIISIL	31.4	-0.3719	Non-antigen			
		ILTMSIMPV	33.0	0.9840	Antigen	Non-allergen	Non-toxin	Yes
		MLIASTLIV	35.3	0.2693	Non-antigen			

		ILTSKHKTY	44.0	1.1168	Antigen	Allergen	Non-toxin	No
		VIIYVFKKI	58.8	-0.0063	Non-antigen			
		VSLSGNLKY	62.2	1.0750	Antigen	Non-allergen	Non-toxin	No
	DRB1_1501	IYVFKKIKM	476.4	0.3446	Non-antigen			
		YSLYRSTCI	572.1	0.5128	Antigen	Allergen	Non-toxin	No
		IISILTMSI	1337.2	0.5157	Antigen	Non-allergen	Non-toxin	Yes
		IIGYDSIIW	1796.2	-0.0988	Non-antigen			
		VLTYSSSIF	2726.1	-0.2037	Non-antigen			
		LIVSLSGNL	2833.6	0.0008	Non-antigen			
		IIYVFKKIK	2951.5	0.5630	Antigen	Allergen	Non-toxin	Yes
		ICMLIASTL	3094.5	0.7125	Antigen	Allergen	Non-toxin	No
	DRB1_1602	WYKYINDRY	1632.7	-0.5812	Non-antigen			
		IYVFKKIKM	2196.9	0.3446	Non-antigen			
		IFRFHSEDI	2976.5	0.2452	Non-antigen			
		YSLYRSTCI	4022.2	0.5128	Antigen	Allergen	Non-toxin	No
		FDRIYNNVV	4943.8	-0.6790	Non-antigen			
		NFINRTFSV	5257.2	0.7245	Antigen	Non-allergen	Non-toxin	No
AIE40738.1	DRB1_0701	IIITVGMLI	90.3	0.8226	Antigen	Allergen	Non-toxin	No
		VIHTNHSDI	148.8	0.8736	Antigen	Non-allergen	Non-toxin	Yes
	DRB1_0801	WKVLSIMAF	269.7	0.9293	Antigen	Allergen	Non-toxin	No
		MLIYSMWGK	765.4	0.1378	Non-antigen			
	DRB1_0901	WKVLSIMAF	536.1	0.9293	Antigen	Allergen	Non-toxin	No
		FSGVLIAGI	784.6	0.1127	Non-antigen			
	DRB1_1001	YFSGVLIAG	868.0	-0.0009	Non-antigen			
		WKVLSIMAF	1617.2	0.9293	Antigen	Allergen	Non-toxin	No
	DRB1_1201	DMMLMIGNY	2034.4	0.7031	Antigen	Non-allergen	Non-toxin	No
		WKVLSIMAF	2203.7	0.9293	Antigen	Allergen	Non-toxin	No
	DRB1_1301	MMLMIGNYF	20.2	0.2284	Non-antigen			
		WKVLSIMAF	58.1	0.9293	Antigen	Allergen	Non-toxin	No

	DRB1_1501	MMLMIGNYF	1643.0	0.2284	Non-antigen			
		MLMIGNYFS	2682.0	0.2211	Non-antigen			
		IFAFIDFSK	3037.9	0.8822	Antigen	Allergen	Non-toxin	Yes
		VLSIMAFIL	4985.4	01959	Non-antigen			
	DRB1_1602	FIDFSKSTS	4193.5	0.9517	Antigen	Non-allergen	Non-toxin	No
AIE40737.1	DRB1_0701	ILYTLYNKI	13.0	0.1086	Non-antigen			
	DRB1_0801	YTLYNKIKN	426.0	0.2588	Non-antigen			
	DRB1_1101	YTLYNKIKN	1378.0	0.2588	Non-antigen			
		LYNLVKSSA	3693.8	-0.1215	Non-antigen			
	DRB1_1201	TVAILYTLY	1758.2	0.5890	Antigen	Allergen	Non-toxin	No
	DRB1_1501	ILYTLYNKI	1699.3	0.1086	Non-antigen			
		VAILYTLYN	1784.4	0.5679	Antigen	Allergen	Non-toxin	No
	DRB1_1602	LYNLVKSSA	5550.4	-0.1215	Non-antigen			
AIE40718.1	DRB1_0101	FRTLLSSSN	82.4	-0.2739	Non-antigen			
		FTYLGTTIN	514.2	1.3088	Antigen	Non-allergen	Non-toxin	No
		ILFLMSQRY	1020.4	1.0469	Antigen	Allergen	Non-toxin	Yes
		LDNLLPSTL	1122.0	0.1797	Non-antigen			
	DRB1_0301	KLNDDTQVY	3195.2	-0.3658	Non-antigen			
		FLMSQRYSR	4187.8	0.9843	Antigen	Non-allergen	Non-toxin	No
		IHSDQLSKF	4561.7	-0.6628	Non-antigen			
		FYLDNLLPS	4731.8	0.2071	Non-antigen			
		LNDDTQVYY	6690.6	-0.1260	Non-antigen			
		IQNTGKLVR	7896.7	0.3230	Non-antigen			
		NIHSDQLSK	9409.4	-0.5227	Non-antigen			
		VYYSGEIIR	9916.7	-0.1812	Non-antigen			
	DRB1_0401	FYLDNLLPS	1845.4	0.2071	Non-antigen			
		YYSGEIIRA	2800.3	-0.0677	Non-antigen			
		YLGTTINHS	3337.8	0.9323	Antigen	Allergen	Non-toxin	No
		FRTLLSSSN	3624.0	-0.2739	Non-antigen			
		IIRAATTSP	5041.0	0.1415	Non-antigen			
		FLMSQRYSR	5207.1	0.9843	Antigen	Non-allergen	Non-toxin	No
		IRAATTSPV	5385.3	0.4958	Antigen	Allergen	Non-toxin	No
		IFLQVSDHK	6707.5	0.3403	Non-antigen			
		FQKIVNQLD	7177.7	-0.3769	Non-antigen			

		YSGEINLVH	7526.1	0.8764	Antigen	Allergen	Non-toxin	No
	DRB1_0701	YNESKPTTI	136.7	0.4770	Antigen	Allergen	Non-toxin	No
	DRB1_0801	FLMSQRYSR	119.2	0.9843	Antigen	Non-allergen	Non-toxin	No
		FSYYQKYIE	200.0	0.6113	Antigen	Allergen	Non-toxin	Yes
		LFLMSQRYS	225.6	0.9976	Antigen	Non-allergen	Non-toxin	Yes
		YVLSTIHIY	702.2	0.5976	Antigen	Allergen	Non-toxin	No
	DRB1_0901	YVLSTIHIY	468.5	0.5976	Antigen	Allergen	Non-toxin	No
		YFTYLGTTI	944.9	1.2121	Antigen	Allergen	Non-toxin	No
		LREACFSYY	1007.0	1.5067	Antigen	Non-allergen	Non-toxin	No
		IRAATTSPV	1083.0	0.4958	Antigen	Allergen	Non-toxin	No
	DRB1_1001	FRTLLSSSN	78.7	-0.2739	Non-antigen			
		FTYLGTTIN	415.5	1.3088	Antigen	Non-allergen	Non-toxin	No
		YFTYLGTTI	576.3	1.2121	Antigen	Allergen	Non-toxin	No
		YYSGEIIRA	697.3	-0.0677	Non-antigen			
		FQKIVNQLD	898.8	-0.3769	Non-antigen			
		LSKFRTLLS	955.7	-0.7350	Non-antigen			
		YLGTTINHS	1264.2	0.9323	Antigen	Allergen	Non-toxin	No
		KFRTLLSSS	1324.9	-0.1537	Non-antigen			
		YFQKIVNQL	1329.9	-0.5633	Non-antigen			
		LKTLDIHYN	1502.0	1.4807	Antigen	Allergen	Non-toxin	Yes
		YISGGFLPN	1563.1	0.5635	Antigen	Non-allergen	Non-toxin	No
	DRB1_1101	FLMSQRYSR	1171.3	0.9843	Antigen	Non-allergen	Non-toxin	No
		LVHWNKKKY	1756.8	1.0410	Antigen	Allergen	Non-toxin	Yes
		LFLMSQRYS	3080.1	0.9976	Antigen	Non-allergen	Non-toxin	Yes
		VHWNKKKYS	4034.1	1.1380	Antigen	Allergen	Non-toxin	Yes
		FRTLLSSSN	4059.2	-0.2739	Non-antigen			

		ILFLMSQRY	4124.7	1.0469	Antigen	Allergen	Non-toxin	Yes
		FYLDNLLPS	4347.3	0.2071	Non-antigen			
	DRB1_1201	ILFLMSQRY	261.9	1.0469	Antigen	Allergen	Non-toxin	Yes
		LREACFSYY	1120.0	1.5067	Antigen	Non-allergen	Non-toxin	No
		RLKTLDIHY	2207.3					
	DRB1_1301	LVHWNKKKY	11.8	1.0410	Antigen	Allergen	Non-toxin	Yes
		ILFLMSQRY	17.0	1.0469	Antigen	Allergen	Non-toxin	Yes
		YVLSTIHIY	30.8	0.5976	Antigen	Allergen	Non-toxin	No
		FLMSQRYSR	31.1	0.9843	Antigen	Non-allergen	Non-toxin	No
		LMSQRYSRE	54.6	0.6987	Antigen	Allergen	Non-toxin	No
		LFLMSQRYS	68.2	0.9976	Antigen	Non-allergen	Non-toxin	Yes
	DRB1_1501	ILFLMSQRY	2239.5	1.0469	Antigen	Allergen	Non-toxin	Yes
		SVFYLDNLL	3080.8	0.0793	Non-antigen			
	DRB1_1602	FMKWLSDLR	2006.8	-0.6057	Non-antigen			
		LSKFRTLLS	2175.9	-0.7350	Non-antigen			
		YFMKWLSDL	2295.9	-0.8350	Non-antigen			
		YFTYLGTTI	2555.2	1.2121	Antigen	Allergen	Non-toxin	No
		FRTLLSSSN	2829.0	-0.2739	Non-antigen			
		WIIFPTPIN	2984.5	0.2427	Non-antigen			
		KFRTLLSSS	3144.8	-0.1537	Non-antigen			
		YFQKIVNQL	3232.6	-0.5633	Non-antigen			
		FLMSQRYSR	4602.0	0.9843	Antigen	Non-allergen	Non-toxin	No
AIE40702.1	DRB1_0101	IKWLPIGLL	1272.2	2.7532	Antigen	Allergen	Non-toxin	No
		DALLPASLK	1444.1	0.6738	Antigen	Allergen	Non-toxin	No
		PIGLLALAI	1555.2	1.2622	Antigen	Non-allergen	Non-toxin	No
	DRB1_0301	IGIDTRLPY	1236.3	1.2256	Antigen	Allergen	Non-toxin	Yes
		IYPDKSIVR	1352.2	-2046	Non-antigen			
	DRB1_0701	IIDIKWLPI	182.3	3.0239	Antigen	Allergen	Non-toxin	No

	DRB1_0901	WLPIGLLAL	532.5	1.9828	Antigen	Allergen	Non-toxin	No
	DRB1_1001	PIGLLALAI	1657.7	1.2622	Antigen	Non-allergen	Non-toxin	No
	DRB1_1201	PIGLLALAI	1327.3	1.2622	Antigen	Non-allergen	Non-toxin	No
		IKWLPIGLL	1858.9	2.7532	Antigen	Allergen	Non-toxin	No
	DRB1_1301	LLALAILIL	34.9	0.6819	Antigen	Allergen	Non-toxin	No
	DRB1_1501	PIGLLALAI	4529.4	1.2622	Antigen	Non-allergen	Non-toxin	No
AIE40669.1	DRB1_0101	FLLLTSIPI	229.8	0.6883	Antigen	Allergen	Non-toxin	Yes
		KHLALARYI	553.4	0.2406	Non-antigen			
		FEFLMSLYK	950.2	0.2590	Non-antigen			
		FSRLKENYI	973.6	0.1656	Non-antigen			
		GLLKKFEFL	1052.7	0.6653	Antigen	Non-allergen	Non-toxin	Yes
		YLMDKLNLT	1086.1	0.8795	Antigen	Non-allergen	Non-toxin	No
		LFYVPGYSI	1510.0	-0.3604	Non-antigen			
		FSRGLSRPL	1589.4	-0.7260	Non-antigen			
	DRB1_0301	VYTDNTKHL	5308.8	0.2162	Non-antigen			
		RILDQETFF	6938.1	-0.1539	Non-antigen			
		TKYDPVLMF	8104.0	0.9791	Antigen	Allergen	Non-toxin	No
		LTKSDVQLL	9990.8	0.5134	Antigen	Allergen	Non-toxin	No
	DRB1_0401	FLLLTSIPI	1437.9	0.6883	Antigen	Allergen	Non-toxin	Yes
		YLMDKLNLT	2568.5	0.8795	Antigen	Non-allergen	Non-toxin	No
		YETLANIST	4342.6	0.2604	Non-antigen			
		YVPGYSITT	5683.5	-0.1792	Non-antigen			
		LFWFKNTQF	5872.5	1.3991	Antigen	Allergen	Non-toxin	No
		WIPETAIWS	7029.0	-0.1739	Non-antigen			
		FIANGSFSG	7627.5	0.4132	Antigen	Allergen	Non-toxin	No
	DRB1_0701	YIFTGPVPI	17.2	0.0600	Non-antigen			
		FLLLTSIPI	36.6	0.6883	Antigen	Allergen	Non-toxin	Yes
		FDITKHTLF	40.4	0.2901	Non-antigen			
		LFYVPGYSI	44.6	-0.3604	Non-antigen			

		FSRLKENYI	51.6	0.1656	Non-antigen			
		LFWFKNTQF	134.0	1.3991	Antigen	Allergen	Non-toxin	No
		FSRGLSRPL	134.9	-0.7260	Non-antigen			
		KHLALARYI	143.9	0.2406	Non-antigen			
	DRB1_0801	FFSRGLSRP	353.2	-0.0321	Non-antigen			
		FLMSLYKGP	366.1	-0.0441	Non-antigen			
		FLLLTSIPI	448.7	0.6883	Antigen	Allergen	Non-toxin	Yes
		LMSLYKGPI	514.9	-0.0191	Non-antigen			
		FWFKNTQFD	755.2	1.7273	Antigen	Allergen	Non-toxin	No
		LALARYIHQ	906.3	0.0365	Non-antigen			
		LLLTSIPIY	915.1	0.3717	Non-antigen			
		TFFSRGLSR	924.9	0.1537	Non-antigen			
	DRB1_0901	FSRGLSRPL	249.8	-0.7260	Non-antigen			
		KHLALARYI	397.6	0.2406	Non-antigen			
		YYPISLGLL	519.3	20.775	Antigen	Non-allergen	Non-toxin	No
		LRKFSALPT	531.1	0.2577	Non-antigen			
		FLLFYVPGY	583.9	-0.3302	Non-antigen			
		FTKYDPVLM	866.5	0.9535	Antigen	Allergen	Non-toxin	No
		YIFTGPVPI	961.3	0.0600	Non-antigen			
		FSRLKENYI	963.6	0.1656	Non-antigen			
		FLLLTSIPI	1321.8	0.6883	Antigen	Allergen	Non-toxin	Yes
	DRB1_1001	FLLLTSIPI	375.8	0.6883	Antigen	Allergen	Non-toxin	Yes
		YETLANIST	510.4	0.2604	Non-antigen			
		YIFTGPVPI	595.0	0.0600	Non-antigen			
		LLTSIPIYN	599.3	0.1333	Non-antigen			
		FEFLMSLYK	675.2	0.2590	Non-antigen			
		LRKFSALPT	752.0	0.2577	Non-antigen			
		YVSYLRKFS	1174.1	-0.0881	Non-antigen			
		FTKYDPVLM	1230.9	0.9535	Antigen	Allergen	Non-toxin	No
		YLRKFSALP	1275.0	0.2869	Non-antigen			
		KFEFLMSLY	1587.5	0.8440	Antigen	Non-antigen	Non-toxin	No
		FISFLLLTS	1664.8	0.7759	Antigen	Non-allergen	Non-toxin	Yes
	DRB1_1101	YVSYLRKFS	344.0	-0.0881	Non-antigen			
		YKNAQRRIL	1731.4	-1.0318	Non-antigen			
		YKPLFSRLK	2221.4	-0.5085	Non-antigen			

		VSYLRKFSA	2860.1	-0.0986	Non-antigen			
		VYKNAQRRI	3147.9	-1.0519	Non-antigen			
		FLLLTSIPI	3418.9	0.6883	Antigen	Allergen	Non-toxin	Yes
		GRYVSYLRK	4358.2	0.5695	Antigen	Allergen	Non-toxin	Yes
	DRB1_1201	LANISTILY	481.5	0.2066	Non-antigen			
		TKHLALARY	485.2	0.5127	Antigen	Non-allergen	Non-toxin	Yes
		KHTLFRYVY	854.4	-0.7528	Non-antigen			
		ITKHTLFRY	890.4	-0.9639	Non-antigen			
		LGLLKKFEF	1442.2	1.1879	Antigen	Non-allergen	Non-toxin	Yes
		FSALPTNEF	1604.7	0.1538	Non-antigen			
		LMSLYKGPI	1961.5	-0.0191	Non-antigen			
		FSRLKENYI	2007.7	0.1656	Non-antigen			
	DRB1_1301	VYKNAQRRI	19.0	-1.0519	Non-antigen			
		ITKHTLFRY	37.2	-0.9639	Non-antigen			
		LFSRLKENY	50.2	0.2524	Non-antigen			
		ISLGLLKKF	56.4	0.8271	Antigen	Non-allergen	Non-toxin	No
	DRB1_1501	LFYVPGYSI	676.5	-0.3604	Non-antigen			
		FLLLTSIPI	1130.2	0.6883	Antigen	Allergen	Non-toxin	Yes
		LFWFKNTQF	1219.0	1.3991	Antigen	Allergen	Non-toxin	No
		YLRKFSALP	1993.1	0.2869	Non-antigen			
		FEFLMSLYK	2766.9	0.2590	Non-antigen			
		LLFYVPGYS	2971.0	-0.4438	Non-antigen			
		TNEFISFLL	2973.2	0.2373	Non-antigen			
		FLLFYVPGY	3412.7	-0.3302	Non-antigen			
		KHLALARYI	3624.5	0.2406	Non-antigen			
		FSRGLSRPL	3737.9	-0.7260	Non-antigen			
		LMSLYKGPI	4260.5	-0.0191	Non-antigen			
		HLALARYIH	4538.6	0.2737	Non-antigen			
		LRKFSALPT	4915.2	0.2577	Non-antigen			
	DRB1_1602	FLLLTSIPI	1139.5	0.6883	Antigen	Allergen	Non-toxin	Yes
		LRKFSALPT	2038.0	0.2577	Non-antigen			
		YETLANIST	2818.0	0.2604	Non-antigen			
		YVPGYSITT	4107.0	-0.1792	Non-antigen			
		WFKNTQFDI	4559.8	1.3512	Antigen	Allergen	Non-toxin	No
		FLLFYVPGY	5035.2	-0.3302	Non-antigen			
		YKPLFSRLK	5406.6	-0.5085	Non-antigen			

Table S2: Prediction of cytotoxic T cell epitopes and their antigenicity, allergenicity, toxicity and interferon-γ inducing ability

Protein ID	Allele	Peptide	Binding Affinity (nM)	Vaxijen score	Antigen/Non-antigen	Allergenicity	Toxicity	Interferon-γ inducing ability
AIE40786.1	HLA-A0101	YTCRGHNYY	7.28	0.8296	Antigen	Non-allergen	Non-toxin	No
		LSLLLFHSY	707.69	0.4192	Antigen	Non-allergen	Non-toxin	No
	HLA-A0201	KMMVRIYFV	2.80	0.4661	Antigen	Allergen	Non-toxin	No
		WIIGLDFGV	11.80	0.7114	Antigen	Non-allergen	Non-toxin	No
		GLDFGVYSI	12.93	1.2732	Antigen	Allergen	Non-toxin	No
		LLLFHSYAI	14.28	0.2825	Non-antigen			
	HLA-A0301	KMRDTLPAK	18.99	1.391	Antigen	Allergen	Non-toxin	No
		ALGEPFSAK	27.99	-0.0205	Non-antigen			
	HLA-A2402	LYAKHYNNI	21.35	0.8813	Antigen	Allergen	Non-toxin	No
		KWLNPVCMF	67.13	0.5717	Antigen	Allergen	Non-toxin	No
		RYKDYVVKW	89.30	0.2387	Non-antigen			
		IYFVSLSLL	126.07	1.4551	Antigen	Non-allergen	Non-toxin	Yes
		YFVSLSLLL	311.90	0.9237	Antigen	Allergen	Non-toxin	No
	HLA-A2601	EITEFFNKM	46.30	-0.2546	Non-antigen			
		WIANYTSKF	175.42	-0.0018	Non-antigen			
	HLA-B0702	LPAKDSKWL	131.22	1.4015	Antigen	Non-allergen	Non-toxin	No

	HLA-B0801	MVRIYFVSL	4.14	1.0205	Antigen	Allergen	Non-toxin	No
		LLLFHSYAI	17.43	0.2825	Non-antigen			
		KMMVRIYFV	148.98	0.4661	Antigen	Allergen	Non-toxin	No
		SNRRYLCTV	163.03	0.4989	Antigen	Non-allergen	Non-toxin	No
	HLA-B2705	RRYLCTVTT	39.72	0.1697	Non-antigen			
		GRYDCYVHY	46.52	0.9012	Antigen	Allergen	Non-toxin	Yes
	HLA-B3901	WKNPSGWII	78.77	-0.5101	Non-antigen			
		NPSGWIIGL	86.86	-0.2291	Non-antigen			
		MKMKMMVRI	440.52	0.8614	Antigen	Allergen	Non-toxin	Yes
	HLA-B4001	FENVTEEYI	102.10	0.1350	Non-antigen			
	HLA-B5801	CSAVSTSLF	35.79	0.2594	Non-antigen			
		QGVVRSHVW	44.37	-0.3723	Non-antigen			
		KTLTTTVVL	68.34	0.1327	Non-antigen			
AIE40778.1	HLA-A0101	VSDYVSELY	2.70	-0.0643	Non-antigen			
		LSCNGETKY	416.18	0.8019	Antigen	Non-allergen	Non-toxin	No
		SCDKNNDQY	490.96	0.1249	Non-antigen			
		VTFTCDSGY	521.20	0.3869	Non-antigen			

		EIESLEATY	823.22	1.1217	Antigen	Allergen	Non-toxin	No
	HLA-A0201	FLISIIVLV	3.05	0.4824	Antigen	Allergen	Non-toxin	No
		TLLCVLPAV	3.77	0.9222	Antigen	Allergen	Non-toxin	No
		TIMGVIFLI	8.53	0.2475	Non-antigen			
		IMALTIMGV	9.49	0.8565	Antigen	Allergen	Non-toxin	Yes
		KMCTVSDYV	14.13	0.4266	Antigen	Allergen	Non-toxin	No
	HLA-A2402	TIMGVIFLI	268.68	0.2475	Non-antigen			
	HLA-A2601	MTINCDVGY	66.78	0.6250	Antigen	Allergen	Non-toxin	No
		EATYHIIIM	562.11	0.4046	Antigen	Allergen	Non-toxin	No
	HLA-B0702	VPTMNNAKL	74.14	0.2287	Non-antigen			
	HLA-B3901	YHSLDPNAV	10.67	1.0653	Antigen	Allergen	Non-toxin	Yes
		YEVNSTMTL	32.49	0.9371	Antigen	Non-allergen	No-toxin	Yes
		MKTISVVTL	63.33	0.8900	Antigen	Allergen	Non-toxin	No
		TCDSGYHSL	136.32	-0.1492	Non-antigen			
		TYHIIIMAL	329.34	0.4999	Antigen	Allergen	Non-toxin	No
		YSFGEYMTI	429.73	0.2069	Non-antigen			
	HLA-B4001	YEVNSTMTL	3.77	0.9371	Antigen	Non-allergen	No-toxin	Yes
		LEATYHIII	11.24	0.6444	Antigen	Allergen	Non-toxin	Yes
		YEVIGVSYI	44.25	1.1025	Antigen	Allergen	Non-toxin	No
		LEHGSCQPV	50.88	0.3503	Non-antigen			

	HLA-B5801	KTISVVTLL	9.79	0.6191	Antigen	Allergen	Non-toxin	No
		VSDYVSELY	49.86	-0.0643	Non-antigen			
		SSTCIDGKW	65.76	0.9705	Antigen	Non-allergen	Non-toxin	No
		NAVCETDKW	73.34	-0.0808	Non-antigen			
		MALTIMGVI	122.02	0.7773	Antigen	Allergen	Non-toxin	No
		YISCTANSW	132.37	0.7152	Antigen	Allergen	Non-toxin	No
AIE40766.1	HLA-A0101	HSEDIELCY	8.31	1.2122	Antigen	Allergen	Non-toxin	
		VSLSGNLKY	529.48	1.0750	Antigen	Non-allergen	Non-toxin	No
		FTAICMLIA	855.02	1.2871	Antigen	Allergen	Non-toxin	No
	HLA-A0201	MMMKWIISI	2.49	-0.0467	Non-antigen			
		ILTMSIMPV	9.05	0.9840	Antigen	Non-allergen	Non-toxin	Yes
		MLIASTLIV	9.20	0.2693	Non-antigen			
	HLA-A0301	LTYSSSIFR	30.06	-0.1911	Non-antigen			
		STSTILTSK	50.90	0.4175	Antigen	Allergen	Non-toxin	No
		IVSLSGNLK	119.48	0.6211	Antigen	Non-allergen	Non-toxin	No
		IIYVFKKIK	120.26	0.5630	Antigen	Allergen	Non-toxin	Yes
	HLA-A2402	TYSSSIFRF	8.91	-0.4436	Non-antigen			
		RYNFINRTF	21.18	0.7428	Antigen	Allergen	Non-toxin	No
		VFFTHGYFL	293.40	0.3009	Non-antigen			

	HLA-A2601	NVFFTHGYF	438.71	0.4356	Antigen	Allergen	Non-toxin	Yes
	HLA-B0801	TSKHKTYSL	28.93	1.2415	Antigen	Allergen	Non-toxin	No
		MMMKWIISI	73.19	-0.0467	Non-antigen			
		MMKWIISIL	91.57	-0.3719	Non-antigen			
		YSLYRSTCI	107.78	0.5128	Antigen	Allergen	Non-toxin	No
		FLKHKYGCS	125.03	0.6659	Antigen	Allergen	Non-toxin	Yes
		LKHKYGCSL	152.80	1.2793	Antigen	Non-allergen	Non-toxin	Yes
	HLA-B2705	YRSTCIAII	77.11	1.6925	Antigen	Non-allergen	Non-toxin	No
		YRYNFINRT	93.75	0.9717	Antigen	Allergen	Non-toxin	Yes
	HLA-B3901	MMMKWIISI	17.10	-0.0467	Non-antigen			
		YRSTCIAII	80.22	1.6925	Antigen	Non-allergen	Non-toxin	No
		LKHKYGCSL	87.11	1.2793	Antigen	Non-allergen	Non-toxin	Yes
		YGCSLNPSL	381.73	0.2511	Non-antigen			
	HLA-B5801	MSIMPVLTY	14.36	0.8206	Antigen	Allergen	Non-toxin	No
		CSVNVSCLI	51.42	0.8331	Antigen	Allergen	Non-toxin	No
		LTMSIMPVL	92.32	1.1100	Antigen	Non-allergen	Non-toxin	No
		VSLSGNLKY	95.05	1.0750	Antigen	Non-allergen	Non-toxin	No
AIE40763.1	HLA-A0101	ITSTKTIEY	26.42	1.1302	Antigen	Allergen	Non-toxin	No
		VSEHFGLLF	203.18	0.6064	Antigen	Allergen	Non-toxin	Yes
		CGDNTGIKY	771.93	-0.3577	Non-antigen			

	HLA-A0201	LLMMTLPSI	5.30	0.4259	Antigen	Allergen	Non-toxin	No
		MLMFIFTGI	5.41	0.8393	Antigen	Non-allergen	Non-toxin	No
		QLAGYILTV	6.01	-0.0246	Non-antigen			
		LLIYLCTFV	8.82	-0.7341	Non-antigen			
		FLFYPSMFT	11.61	0.2117	Non-antigen			
		MTLPSIFLI	14.85	0.3971	Non-antigen			
		KLSCAVHLI	16.08	0.4933	Antigen	Allergen	Non-toxin	No
		IMVSEHFGL	30.63	0.8002	Antigen	Allergen	Non-toxin	
		SLSDSDVSL	33.78	1.2532	Antigen	Allergen	Non-toxin	Yes
		FTLRHIIGL	37.67	0.7181	Antigen	Non-allergen	Non-toxin	No
		LLLSGLGTI	39.11	0.3842	Non-antigen			
	HLA-A0301	VLGLGLSLK	45.31	1.8428	Antigen	Allergen	Non-toxin	Yes
		TSKTTILSK	124.87	0.5891	Antigen	Allergen	Non-toxin	No
	HLA-A2402	YYQLAGYIL	27.45	0.1599	Non-antigen			
		IYLCTFVVI	30.91	-0.3139	Non-antigen			
		AYTSISVVF	34.61	1.1050	Antigen	Non-allergen	Non-toxin	Yes
		DYQTMLMFI	79.85	0.5356	Antigen	Non-allergen	Non-toxin	No
		LMMTLPSIF	121.93	0.1923	Non-antigen			

		IYYQLAGYI	134.77	0.2631	Non-antigen			
		AFLFYPSMF	153.90	0.1614	Non-antigen			
		YQTMLMFIF	215.54	0.3973	Non-antigen			
		LFYPSMFTL	233.58	0.2696	Non-antigen			
		IFLIITKVF	300.78	0.2382	Non-antigen			
	HLA-A2601	MVSEHFGLL	87.04	0.7807	Antigen	Allergen	Non-toxin	No
		FIFTGITLF	242.37	0.8996	Antigen	Allergen	Non-toxin	No
		ELCGAFLFY	353.83	-0.647	Non-antigen			
		IIYYQLAGY	481.81	0.3581	Non-antigen			
		CIIDNPTKY	658.51	-0.5458	Non-antigen			
		CAVHLIIYY	969.92	-0.1357	Non-antigen			
	HLA-B0801	MLRVRILLI	14.86	0.7831	Antigen	Allergen	Non-toxin	Yes
		WLLCKLSCA	49.90	0.3963	Non-antigen			
		MLMFIFTGI	106.23	0.8393	Antigen	Non-allergen	Non-toxin	No
		LLMMTLPSI	156.26	0.4259	Antigen	Allergen	Non-toxin	No
		LIITKVFSF	219.74	0.3177	Non-antigen			
	HLA-B2705	LRHIIGLLM	21.11	-0.2650	Non-antigen			

		LRVRILLIY	50.71	0.7118	Antigen	Allergen	Non-toxin	Yes
	HLA-B3901	TKVFSFWLL	67.59	0.0557	Non-antigen			
		YYQLAGYIL	85.52	0.1599	Non-antigen			
		EHFGLLFLV	109.52	0.9194	Antigen	Non-allergen	Non-toxin	Yes
		WKLDNHDIL	117.77	0.4789	Antigen	Non-allergen	Non-toxin	No
		SMFTLRHII	208.57	0.5652	Antigen	Allergen	Non-toxin	No
		VHLIIYYQL	333.84	0.4360	Antigen	Non-allergen	Non-toxin	Yes
		HTSARLHSL	400.69	0.4810	Antigen	Allergen	Non-toxin	No
		MFIFTGITL	417.15	1.1652	Antigen	Allergen	Non-toxin	No
		TVLGLGLSL	452.64	1.3036	Antigen	Allergen	Non-toxin	No
		NKTSKTTIL	465.70	0.2606	Non-antigen			
	HLA-B4001	SEHFGLLFL	11.44	0.9469	Antigen	Non-allergen	Non-toxin	Yes
		KECVDGTLL	22.73	0.2449	Non-antigen			
		IELCGAFLF	25.37	-0.4283	Non-antigen			
	HLA-B5801	MTLPSIFLI	6.44	0.3971	Non-antigen			
		IITKVFSFW	34.04	-0.3022	Non-antigen			
		LSCAVHLII	37.23	0.0829	Non-antigen			
		GTIMVSEHF	50.72	0.2432	Non-antigen			

		KLIQRHTNW	73.44	0.9000	Antigen	Allergen	Non-toxic	No
AIE40739.1	HLA-A0201	FIWFIFHFV	4.34	0.8683	Antigen	Non-allergen	Non-toxin	Yes
		FIFHFVYSA	5.43	0.5524	Antigen	Allergen	Non-toxin	Yes
		LLLVITCCV	32.77	-0.1927	Non-antigen			
	HLA-A2402	VFIWFIFHF	59.51	0.4355	Antigen	Allergen	Non-toxin	No
		IFAVQTIVF	241.14	0.4262	Antigen	Non-allergen	Non-toxin	No
	HLA-B3901	TKIDIIFAV	56.84	1.4032	Antigen	Allergen	Non-toxin	No
	HLA-B5801	AVQTIVFIW	43.37	0.0882	Non-antigen			
		ISNYEPLLL	97.77	0.9456	Antigen	Allergen	Non-toxin	Yes
		QTIVFIWFI	136.42	0.3869	Non-antigen			
AIE40738.1	HLA-A0201	FILGIIITV	4.64	0.6486	Antigen	Allergen	Non-toxin	No
		SIMAFILGI	8.60	0.2928	Non-antigen			
		VLIAGIILL	11.62	0.2172	Non-antigen			
		ILSCIFAFI	17.97	-0.3964	Non-antigen			
		KVLSIMAFI	29.00	0.2618	Non-antigen			
	HLA-A0301	MLIYSMWGK	19.52	0.1378	Non-antigen			
		STSPTRTWK	96.70	-0.0041				
	HLA-A2601	DMMLMIGNY	900.70	0.7031	Antigen	Non-allergen	Non-toxin	No
	HLA-B0702	APHRVSGVI	9.32	0.4917	Antigen	Non-allergen	Non-toxin	No

		SPTRTWKVL	11.17	-0.3057	Non-antigen			
	HLA-B0801	MIGNYFSGV	228.85	-0.3344	Non-antigen			
	HLA-B3901	WKVLSIMAF	407.09	0.9293	Antigen	Allergen	Non-toxin	No
	HLA-B5801	KSTSPTRTW	4.38	-0.0037	Non-antigen			
		RTWKVLSIM	47.50	0.6901	Antigen			
		KVLSIMAFI	132.17	0.2618	Non-antigen	Allergen	Non-toxin	No
		VGMLIYSMW	136.54	0.5505	Antigen	Allergen	Non-toxin	No
AIE40737.1	HLA-A0201	LLIGICVAV	12.05	0.2696	Non-antigen			
	HLA-A0301	AILYTLYNK	51.39	0.4294	Antigen	Allergen	Non-toxin	Yes
	HLA-A2601	TVAILYTLY	220.38	0.5890	Antigen	Allergen	Non-toxin	No
	HLA-B3901	DHISSLYNL	177.18	0.4058	Antigen	Allergen	Non-toxin	No
	HLA-B4001	LEKDHISSL	205.35	0.3818	Non-antigen			
	HLA-B5801	VAVTVAILY	30.36	0.8124	Antigen	Allergen	Non-toxin	No
		VTVAILYTL	110.25	0.6745	Antigen	Allergen	Non-toxin	No
AIE40718.1	HLA-A0101	ITENYRNPY	12.32	0.8011	Antigen	Non-allergen	Non-toxin	No
		LNDDTQVYY	57.91	-0.1260	Non-antigen			
		VSDHKNVYF	197.66	1.0903	Antigen	Allergen	Non-toxin	No
		LSDLREACF	349.90	1.7255	Antigen	Non-allergen	Non-toxin	No
		TTSPVRENY	516.23	0.7917	Antigen	Non-allergen	Non-toxin	No

		GSNHLIDVY	702.32	-0.0002	Non-antigen			
	HLA-A0201	FILTAILFL	8.31	0.5392	Antigen	Allergen	Non-toxin	No
		YLDNLLPST	10.19	0.2106	Non-antigen			
		STLDYFTYL	13.58	0.1219	Non-antigen			
	HLA-A2402	KYIEGNKTF	110.27	-0.5044	Non-antigen			
		YFMKWLSDL	116.62	-0.8350	Non-antigen			
		VFILTAILF	125.39	0.3203	Non-antigen			
		TFAIIAIVF	195.98	1.1105	Antigen	Allergen	Non-toxin	No
		KYSGEINLV	244.94	0.5484	Antigen	Allergen	Non-toxin	No
	HLA-A2601	YVLSTIHIY	176.47	0.5976	Antigen	Allergen	Non-toxin	No
		TTIQNTGKL	345.22	0.6904	Antigen	Non-allergen	Non-toxin	No
		QVSDHKNVY	693.24	0.2986	Non-antigen			
		DLREACFSY	948.20	2.2258	Antigen	Non-allergen	Non-toxin	No
	HLA-B3901	YKYSGEINL	84.64	0.4526	Antigen	Allergen	Non-toxin	No
		IRAATTSPV	91.42	0.4958	Antigen	Allergen	Non-toxin	No
		HSADAAWII	156.49	0.8212	Antigen	Non-allergen	Non-toxin	No
		FVFILTAIL	174.39	0.4639	Antigen	Allergen	Non-toxin	Yes
		KHDDGIIII	240.33	0.4287	Antigen	Non-allergen	Non-toxin	Yes
		NHEGKPHYI	502.18	0.6450	Antigen	Allergen	Non-toxin	Yes

	HLA-B4001	RENYFMKWL	30.82	0.0580	Non-antigen			
	HLA-B5801	MSAPFDSVF	11.72	-0.0184	Non-antigen			
		RSANMSAPF	19.40	0.9595	Antigen	Allergen	Non-toxin	Yes
		HSADAAWII	82.22	0.8212	Antigen	Non-allergen	Non-toxin	No
		VLSTIHIYW	92.80	1.2426	Antigen	Allergen	Non-toxin	No
AIE40702.1	HLA-A0101	ATENIAVRY	22.82	0.4092	Antigen	Non-allergen	Non-toxin	Yes
		MTDEQIYAF	35.33	0.1515	Non-antigen			
		GIDTRLPYY	108.19	0.8200	Antigen	Allergen	Non-toxin	
	HLA-A0201	GLLALAILI	39.83	0.7314	Antigen	Allergen	Non-toxin	
	HLA-A0301	ALLPASLKK	21.42	0.7775	Antigen	Non-allergen	Non-toxin	
	HLA-A2402	RYPIIDIKW	102.23	2.5629	Antigen	Non-allergen	Non-toxin	Yes
	HLA-B3901	EPCKRSDAL	245.79	0.5507	Antigen	Non-allergen	Non-toxin	No
	HLA-B4001	TENIAVRYL	100.29	0.2487	Non-antigen			
	HLA-B5801	LAILILAFF	106.82	0.4166	Antigen	Allergen	Non-toxin	No
AIE40669.1	HLA-A0101	YTDNTKHLA	19.63	0.4447	Antigen	Allergen	Non-toxin	No
		ETAIWSSRY	20.68	0.4582	Antigen	Non-allergen	Non-toxin	Yes
		LANISTILY	219.42	0.2066	Non-antigen			
		YANLDASDY	771.39	0.9525	Antigen	Non-allergen	Non-toxin	No
	HLA-A0201	VLMFLLFYV	2.39	0.2059	Non-antigen			

		SLYKGPIPV	4.67	-0.0538	Non-antigen			
		YLMDKLNLT	4.73	0.8795	Antigen	Non-allergen	Non-toxin	No
		YIFTGPVPI	6.44	0.0600	Non-antigen			
		FLLLTSIPI	8.95	0.6883	Antigen	Allergen	Non-toxin	Yes
		YLFDNYAYG	30.91	0.0523	Non-antigen			
	HLA-A0301	RGLSRPLMK	120.30	-0.0789	Non-antigen			
	HLA-A2402	RYVSYLRKF	25.60	-0.1696	Non-antigen			
		IWSSRYANL	74.79	0.9676	Antigen	Allergen	Non-toxin	No
		PLMKNTYLF	78.61	0.2512	Non-antigen			
		YYPISLGLL	86.33	2.0775	Antigen	Non-allergen	Non-toxin	No
		SYLRKFSAL	109.36	-0.2127	Non-antigen			
	HLA-A2601	ETAIWSSRY	4.90	0.4582	Antigen	Non-allergen	Non-toxin	Yes
		STILYFTKY	90.62	0.4048	Antigen	Non-allergen	Non-toxin	No
	HLA-B0702	RPLMKNTYL	13.91	0.1050	Non-antigen			
	HLA-B0801	SYLRKFSAL	34.12	-0.2127	Non-antigen			
		RPLMKNTYL	62.23	0.1050	Non-antigen			
		GLLKKFEFL	176.20	0.6653	Antigen	Non-allergen	Non-toxin	Yes
	HLA-B2705	GRYVSYLRK	39.73	0.5695	Antigen	Allergen	Non-toxin	Yes

		RRILDQETF	79.94	-0.9809	Non-antigen			
	HLA-B3901	HQTGDYKPL	69.56	0.6749	Antigen	Allergen	Non-toxin	No
		YIFTGPVPI	129.67	0.0600	Non-antigen			
		YKNAQRRIL	147.81	-1.0318	Non-antigen			
		FLLLTSIPI	155.22	0.6883	Antigen	Allergen	Non-toxin	Yes
		KKFEFLMSL	304.14	1.0621	Antigen	Allergen	Non-toxin	No
		TSIPIYNIL	389.05	0.2089	Non-antigen			
	HLA-B4001	NEFISFLLL	17.45	0.4482	Antigen	Allergen	Non-toxin	Yes
		QETFFSRGL	29.68	0.3990	Non-antigen			
		TEFIANGSF	29.87	0.4011	Antigen	Non-allergen	Non-toxin	Yes
		YEEKVNTEF	30.31	0.6875	Antigen	Non-allergen	Non-toxin	No
	HLA-B5801	LANISTILY	87.02	0.2066	Non-antigen			

Table S3: Prediction of B cell epitopes and their antigenicity, allergenicity, toxicity and interferon-γ inducing ability

Protein ID	Peptide	Vaxijen	Antigen/Non-antigen	Allergen	Toxin	Interferon
>AIE40786.1	TLPAKDSKW	1.7428	Antigen	Allergen	Non-toxin	No
	PFSAKCPPIE	1.3134	Antigen	Non-allergen	Non-toxin	Yes
	VTIGEPANI	0.9400	Antigen	Allergen	Non-toxin	No
AIE40778.1	NAKLTSTETSFNDK	1.4377	Antigen	Non-allergen	Non-toxin	No
	GYHSLDPNAVCETDKWKYENP	0.5582	Antigen	Allergen	Non-toxin	Yes
	EKNGNTSWNDTVTCPNAEC	0.8791	Antigen	Non-allergen	Non-toxin	No
	GSCQPVKEKYS	0.3830	Non-antigen			
	TLTGSPSSTCIDG	0.3234	Non-antigen			
	RSNEEFDPVDDGPDDETDLSK	0.7255	Antigen	Allergen	Non-toxin	No
AIE40766.1	None					
AIE40763.1	TYTCGDNTGI	-0.4193	Non-antigen			
AIE40739.1	None					
AIE40738.1	None					
AIE40737.1	IKNPQNPNPSPNLNSPPPETRNTK	0.7588	Antigen	Non-allergen	Non-toxin	Yes
AIE40718.1	NESKPTTIQN	0.3613	Non-antigen			
	KKYSSYEEAKKHD	0.4567	Antigen	Allergen	Non-toxin	No
	SNHEGKPHYITENYRNPYKLNDDTQVY	0.2647	Non-antigen			
AIE40702.1	None					

AIE40669.1	IDHPNLSRARSPS DYET	0.1146	Non-antigen			

Table S4: Physiochemical Properties of the MPXV-MEV construct

Number of amino acids	390
Molecular weight	40293.29
Antigenicity	Antigenic (Vaxijen score: 0.5492)
Allergic potential	Non-allergen
Theoretical pI	9.75
Instability index	Stable (36.04)
Total number of atoms	5682
Aliphatic index	92.72
Extinction coefficient	36565
Grand average of hydropathicity (GRAVY)	-0.009
Estimated half-life	>20 hours in yeast, >10 hours in E. coli, and 30 hours in mammalian reticulocytes
